# Prehospital use of a modified HEART Pathway and point-of-care troponin to predict cardiovascular events

**DOI:** 10.1371/journal.pone.0239460

**Published:** 2020-10-07

**Authors:** Jason P. Stopyra, Anna C. Snavely, Lane M. Smith, R. David Harris, Robert D. Nelson, James E. Winslow, Roy L. Alson, Gregory J. Pomper, Robert F. Riley, Nicklaus P. Ashburn, Nella W. Hendley, Jeremiah Gaddy, Tyler Woodrum, Louis Fornage, David Conner, Manrique Alvarez, Adam Pflum, Lauren E. Koehler, Chadwick D. Miller, Simon A. Mahler

**Affiliations:** 1 Department of Emergency Medicine, Wake Forest University School of Medicine, Winston-Salem, North Carolina, United States of America; 2 Department of Biostatistics, Wake Forest University School of Medicine, Winston-Salem, North Carolina, United States of America; 3 Forsyth County Emergency Services, Forsyth County Government, Winston-Salem, North Carolina, United States of America; 4 Department of Pathology, Wake Forest University School of Medicine, Winston-Salem, North Carolina, United States of America; 5 Department of Cardiology, The Christ Hospital Heart and Vascular Center, Cincinnati, Ohio, United States of America; 6 Department of Emergency Medicine, Washington University School of Medicine, St. Louis, Missouri, United States of America; 7 Department of Emergency Medicine, Duke University, Durham, North Carolina, United States of America; 8 Department of Cardiology, Wake Forest University School of Medicine, Winston-Salem, North Carolina, United States of America; UMCU, NETHERLANDS

## Abstract

The HEART Pathway is a validated risk stratification protocol for Emergency Department patients with chest pain that has yet to be tested in the prehospital setting. This study seeks to test the performance of a prehospital modified HEART Pathway (PMHP). A prospective cohort study of adults with chest pain without ST-segment elevation myocardial infarction was conducted at three EMS agencies between 12/2016-1/2018. To complete a PMHP assessment, paramedics drew blood, measured point-of-care (POC) troponin (i-STAT; Abbott Point of Care) and calculated a HEAR score. Patients were stratified into three groups: high-risk based on an elevated troponin, low-risk based on a HEAR score <4 with a negative troponin, or moderate risk for a HEAR score ≥4 with a negative troponin. Sensitivity, specificity, negative and positive predictive values of the PMHP for detection of major adverse cardiac events (MACE: cardiac death, MI, or coronary revascularization) at 30-days were calculated. A total of 506 patients were accrued, with PMHP completed in 78.1% (395/506). MACE at 30-days occurred in 18.7% (74/395). Among these patients, 7.1% (28/395) were high risk yielding a specificity and PPV for 30-day MACE of 96.6% (95%CI: 94.0–98.3%) and 60.7% (95%CI: 40.6–78.6%) respectively. Low-risk assessments occurred in 31.4% (124/395), which were 90.5% (95%CI: 81.5–96.1%) sensitive for 30-day MACE with a NPV of 94.4% (95%CI: 88.7–97.7%). Moderate-risk assessments occurred in 61.5% (243/395), of which 20.6% had 30-day MACE. The PMHP is able to identify high-risk and low-risk groups with high specificity and negative predictive value for 30-day MACE.

**Clinical trial registration:**

clinicaltrials.gov (NCT02709135).

## Introduction

Chest pain is a common reason for emergency medical services (EMS) activation, and nearly half of all patients with acute coronary syndrome (ACS) come to the Emergency Department (ED) via ambulance [1, 2]. The prehospital assessment of patients with chest pain is largely focused on the detection of ST-segment elevation myocardial infarction (STEMI) via prehospital 12-lead electrocardiogram (ECG). However, STEMIs are present in only seven percent of patients with chest pain transported to the hospital by EMS [3, 4]. Prehospital care for the remaining patients, with chest pain and without STEMI, is driven by treatment protocols and destination plans that are largely agnostic regarding patients’ short-term risk for other adverse cardiac events. Thus, in patients without STEMI, EMS providers use gestalt and patient preference, rather than a structured risk assessment to determine prehospital transport destination. High-risk patients are often transported to facilities without interventional cardiology capabilities, and those who are later found to have non-STEMI ACS, require subsequent transfer for urgent revascularization [5]. Furthermore, low-risk patients are often transported directly to crowded tertiary care facilities, despite not needing advanced cardiac imaging capabilities or interventional cardiology. Translation of risk stratification tools validated in the ED setting to the prehospital setting for patients with possible ACS, but without STEMI, could prevent costly and inefficient transfers and avoid crowding of tertiary care center EDs [6, 7]. Our investigative team has previously demonstrated that the History, ECG, Age, Risk Factors, and Troponin (HEART) Pathway is safe and effective in the ED setting [8–14]. However, the HEART Pathway has not been studied in the prehospital setting. A prehospital HEART Pathway modified for prehospital use and paired with in-ambulance quantitative point of care (POC) troponin (cTn) measurement has the potential to improve the accuracy of prehospital triage, allowing paramedics to tailor care delivery and destination plans for patients with acute chest pain based on an objective risk assessment. Furthermore, only a few studies have evaluated quantitative POC cTn testing in the prehospital environment and none have integrated POC testing into a diagnostic pathway [15–20]. Therefore, the objective of this study is to establish the 30-day prognostic performance of a prehospital modified HEART Pathway (PMHP) assessment including a POC cTn measurement for identifying patients with short-term adverse cardiovascular events.

## Materials and methods

We conducted a prospective cohort study within three EMS systems from December 2016 to January 2018. Over 150 paramedics were trained to use the i-STAT device (Abbott Point of Care, Princeton NJ, USA) for POC cTn measurement and to calculate a HEAR score. In this study, the PMHP (HEAR score assessment including POC cTn measurement) results were not used clinically to alter treatment or destination protocols. Study blood collection was within the scope of practice of paramedics, who routinely perform venipuncture and conduct POC testing (i.e. blood glucose) on acutely ill patients. This study was performed under a waiver of informed consent obtained from the Wake Forest University School of Medicine Institutional Review Board, conducted in compliance with STARD guidelines and was registered with clinicaltrials.gov (NCT02709135) prior to patient accrual.

This study was conducted in three central North Carolina county EMS agencies. Forsyth County EMS, an urban agency has approximately 80 paramedics and 16 ambulances, completes about 35,000 patient transports annually. Stokes County EMS, a rural agency, has 34 paramedics, 5 ambulances, and completes 6,000 transports each year. Surry EMS, also a rural agency, has 73 paramedics, 7 ambulances, and completes approximately 17,000 transports annually. Participation was limited to patients transported to Wake Forest Baptist Medical Center (WFBMC), the coordinating medical center ED. WFBMC is a tertiary care center, level 1 trauma center for adults and pediatrics with 821 licensed beds, full specialty/subspecialty availability, and full cardiac catheterization lab capability. The ED has 47 beds with an annual volume of approximately 105,000 visits. The ED is staffed by board certified or board eligible emergency physicians 24 hours per day, 7 days a week who directly provide care and oversee care provided by residents and advance practice providers.

**Fig 1:**
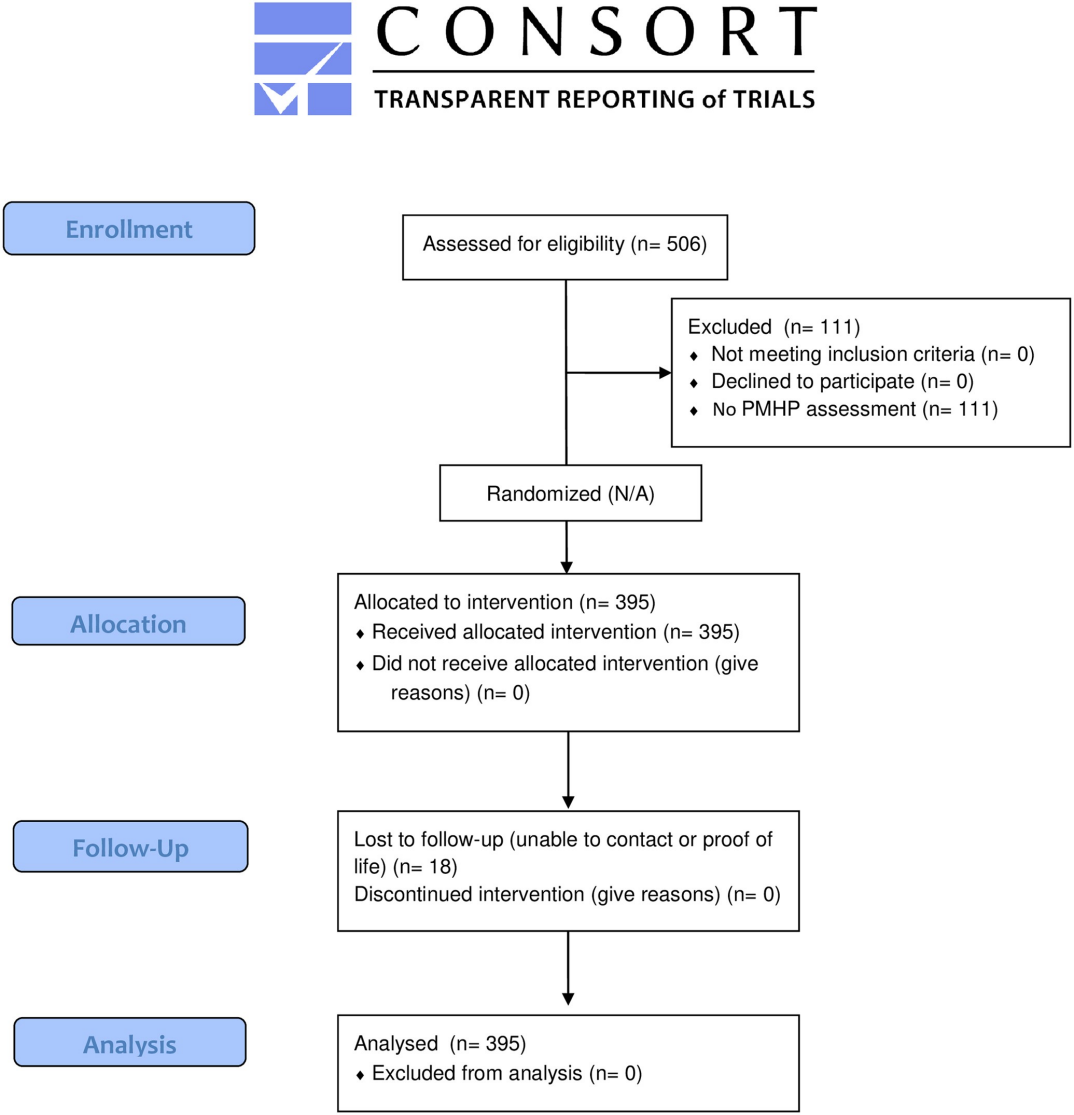
CONSORT 2010 flow diagram.

The target population was a convenience sample of adult patients ≥21 years old with acute, non-traumatic chest pain and without evidence of STEMI on ECG (ST-segment elevation in contiguous leads on any electrocardiogram (≥ 1 mV) transported to the coordinating medical center. Patients being transferred from other acute care facilities, those in which a blood sample could not be obtained, and those with scene and transportation times anticipated to be less than 5 minutes were excluded. Patients with concomitant non-cardiac medical, surgical, or psychiatric emergencies, those receiving hospice care, and patients with unstable vital signs; symptomatic hypotension (systolic blood pressure < 90 mm Hg), tachycardia (heart rate>120), bradycardia (heart rate<40), and hypoxemia (<90% pulse-oximetry on room air or normal home oxygen flow rate) were also excluded. The CONSORT flow diagram is presented in Fig 1.

Paramedics were trained to identify subjects appropriate for inclusion and to calculate a HEAR score through in-person teaching as well as self-learning video refresher modules. This training included identification of non-specific ECG changes and use of the computer monitor ECG analysis to assist in this. Training regarding use of the i-STAT device was conducted by the manufacturer’s trainers. This included the proper storage, maintenance, calibration, use, and the interpretation and reporting of results.

**Fig 2:**
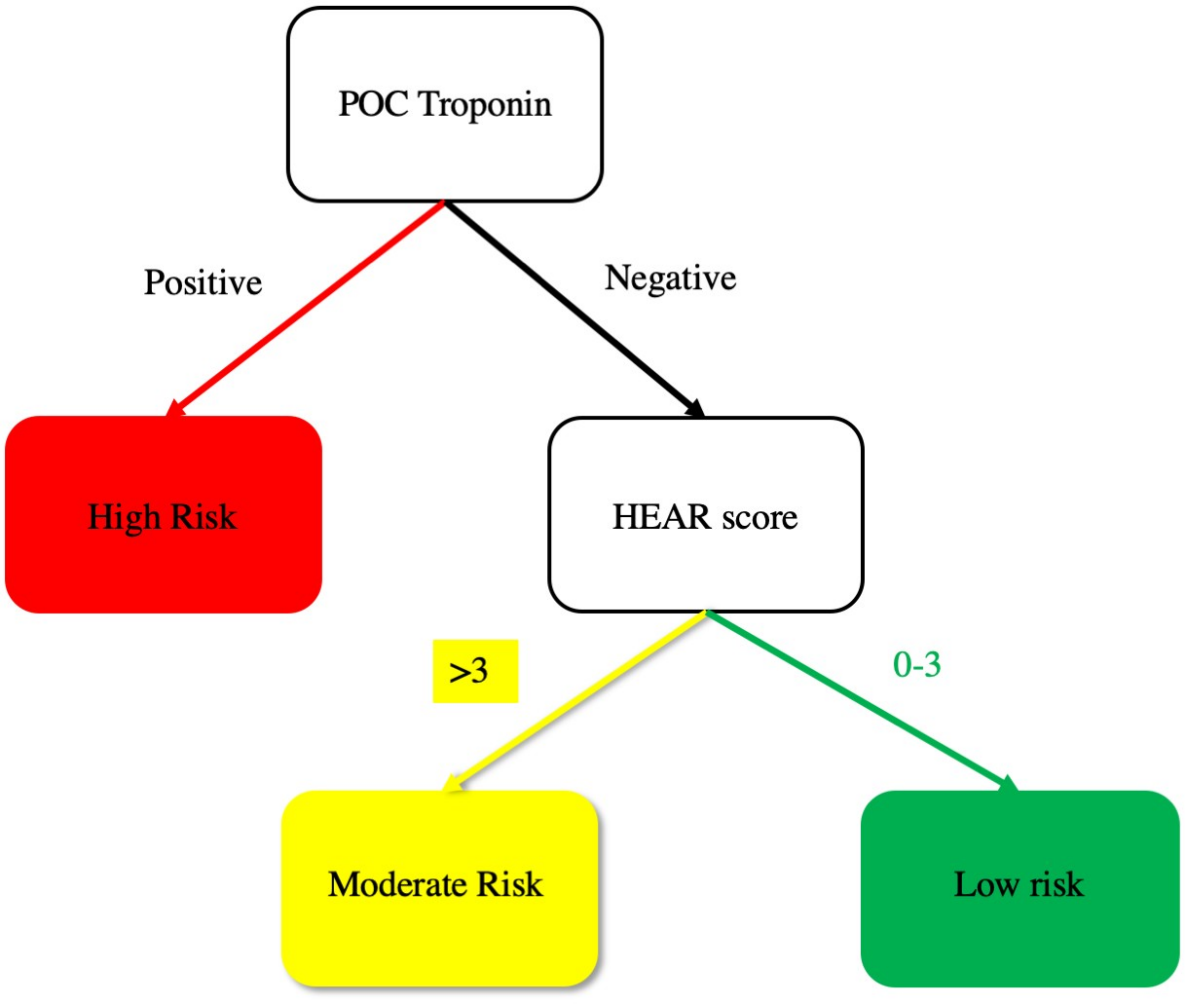
Prehospital modified HEART Pathway.

Following training, 18 ambulances across the three agencies were equipped with an i-STAT device, blood collection supplies, and a room-temperature cooler (Koolatron; Ontario, Canada) to store reagents. In eligible patients, paramedics collected blood into a single Lithium Heparin tube. This prehospital blood sample was used to measure POC cTn during patient transport on an i-STAT device secured to the paramedic’s workstation in the ambulance, which is an off-label use of the device. An i-STAT POC cTn measurement greater than or equal to the 99^th^ percentile upper reference limit (URL), which was 0.080 ng/ml, was considered positive and consistent with a high-risk short-term prognosis based on the PMHP assessment (see Fig 2). Remaining prehospital blood was stored for core lab measurement on arrival to WFBMC. Paramedics used a worksheet to obtain the historical and clinical data needed to calculate a HEAR score. The HEAR score has four components: History, Electrocardiogram (ECG), Age, and Risk factors [21, 22]. Patients with a HEAR score <4 and a cTn measurement less than the 99^th^ percentile of the URL were considered low-risk. Consistent with data from the HEART Pathway Implementation Study, if the HEAR score was ≥4 with a cTn measurement less than the URL the patient was considered moderate risk (See Fig 2) [10, 23]. To assess the short-term prognostic performance of the PMHP using a more sensitive troponin assay, paramedic HEAR scores were also combined with a core lab measurement of cTn using the blood sample collected in the ambulance. This combination was used to create a core-lab-PMHP assessment. A patient was considered high risk by core-lab-PMHP if the core-lab cTn measurement was greater than or equal to the URL [AccuTnI+3 assay (Beckman Coulter, California) URL; 0.025 ng/L or TnI-Ultra assay (Siemens, Munich Germany) URL; 0.040 ng/L]. A low-risk assessment by the core-lab-PMHP required a HEAR score <4 and a core-lab cTn measurement less than the URL. Patients with a HEAR score ≥4 and core-lab cTn less than the URL were classified as moderate risk by the core-lab-PMHP.

Protocol driven routine chest pain care, triage and destination plans for participating EMS counties included obtaining intravenous access, an ECG, and the administration of aspirin, nitroglycerin, and supplemental oxygen. The paramedics were instructed that the PMHP assessment as well as individual POC cTn and HEAR score results were not to be used to alter the patient’s transportation, destination, or care. Paramedics were not blinded to POC cTn results and therefore were not prevented from sharing their results with the ED care team as part of their typical transfer of care reports. This transfer of information was done using a study specific form, which clearly stated that the POC troponin value was for research purposes only and was not to be used to alter a patient’s clinical care. The ED care team received report on the POC cTn results, the patient’s clinical features and ECG as part of a normal EMS verbal report, but the ED care team remained blinded to paramedics’ HEAR scores.

While in the ED, participants received a standard ED chest pain evaluation including an ECG, serologic studies, and independent use of the HEART Pathway risk stratification decision support tool. As part of normal care, blood obtained in the ED at presentation and 3 hours following presentation were used for cTn measurement using the coordinating medical center’s core laboratory [AccuTnI+3 assay (4/3/17-12/12/17) or TnI-Ultra assay (before 4/3/17 and after 12/12/17)].

The primary objective of this project was to evaluate the prognostic performance (i.e., sensitivity, specificity, positive and negative predictive values and positive and negative likelihood ratios) of a PMHP assessment to predict 30-day major adverse cardiac events (MACE). A secondary objective was to assess the prognostic performance for the PMHP for cardiac death or MI at 30-days. Medical record review and phone follow-up was conducted for each patient 30–90 days following their pre-hospital encounter to screen for MACE events within the 30-day study period.

MACE and its components were target conditions evaluated during the index visit and through 30 days of follow-up. Cardiac death was based on the reference standards defined in the modified Action to Control Cardiovascular Risk in Diabetes (ACCORD) trial [24]. MI was defined using the reference standard within the Joint European Society of Cardiology/American College of Cardiology Foundation/American Heart Association/World Health Federation Task Force universal definition [25]. All components of the primary MACE composite were adjudicated by three cardiovascular experts (two primary reviewers and one secondary reviewer). Any discrepancies among the two primary reviewers were resolved by the third reviewer. Participants without follow up data were searched for in the North Carolina Death Index (NCDI). Those not found in the NCDI were considered free from adverse events.

This study was powered to detect a sensitivity of 98% with a lower bound of the 95% confidence interval exceeding 90% for the PMHP assessment for 30-day MACE. To achieve this, fifty patients with MACE were needed. Assuming a 10% MACE rate among our chest pain population [5], a total sample size of 500 patients was determined.

Among patients with completed assessments, the percentage of patients identified by paramedics as high, moderate, or low risk (see Fig 2) and the percentage of patients with each component of the HEAR score were calculated. Measures of diagnostic performance for the PMHP were calculated for both MACE and death/MI at 30 days (inclusive of index visit events). Since the PMHP has three risk categories two different sets of 2x2 tables were constructed to evaluate diagnostic performance for each outcome: high risk vs. moderate or low risk (denoted as high risk assessment in the results) and high or moderate risk vs. low risk (denoted as low risk assessment in the results). Specificity, positive predictive value (PPV), and positive likelihood ratio (+LR) were reported for high risk assessments, as these are most relevant when comparing high risk to non-high risk and sensitivity, negative predictive value (NPV) and negative likelihood ratio (-LR) were calculated for low risk assessments, as these are most relevant when comparing non-low risk to low risk. Sensitivity, specificity, PPV and NPV were reported along with exact 95% confidence intervals and +LR and -LR were reported along with confidence intervals calculated using the method of Simel et al. [26]. Diagnostic characteristics for the core-lab-PMHP (core-lab cTn measure of prehospital blood combined with paramedic HEAR score) were also evaluated using the same approach as described for PHMP. Receiver operating curves were also generated as a way to visualize the diagnostic characteristics for both the PHMP and core-lab-PHMP assessments. Patient characteristics were summarized and compared between those with and without 30-day MACE using Wilcoxon rank-sum or Fisher’s exact tests. Analyses were performed using SAS 9.4 (SAS Institute, Cary, North Carolina) or R 3.5.1 (www.R-project.org).

## Results

From 12/2016-4/2018, three EMS agencies accrued 506 eligible patients. PMHP assessments were completed by paramedics in 78.1% (395/506) of patients. Patient characteristics in the cohort and among those with and without 30-day MACE are described in Table 1. Characteristics of the 395 patients with PMHP assessments were similar to those without assessments (S1 Table). The frequency of PMHP components are summarized in Table 2. Among patients with completed PMHP assessments 18.7% (74/395) had 30-day MACE; with 0 deaths, 70 MIs, and 4 revascularization events without MI. Of the 4.6% (18/395) of patients lost to follow up none were found in the North Carolina Death Index.

**Table 1:** PARAHEART patient characteristics (30-day MACE).

Patient Characteristic	Total	Patients with MACE n = 74	Patients without MACE n = 321	MACE vs. no MACE
	N = 395			
Age years–mean ± SD	58.5 ± 15.2	64.4 ± 13.1	57.2 ± 15.4	**P <0.0001**
Sex (female)	210/395 (53.2%)	31/74 (41.9%)	225/321 (53.4%)	**P = 0.038**
Race				
Caucasian	199/392 (50.8%)	39/73 (53.4%)	160/319 (50.2%)	**P = 0.023**
African American	173/392 (44.1%)	28/73 (38.4%)	145/319 (45.5%)	
Asian	3/392 (0.8%)	3/73 (4.1%)	0/319 (0%)	
Native American	1/392 (0.3%)	0/73 (0%)	1/319 (0.3%)	
Other	16/392 (4.1%)	4/73 (4.8%)	13/319 (4.1%)	
Ethnicity (Hispanic)	16/395 (4.1%)	2/74 (2.7%)	14/321 (4.4%)	P = 0.747
Risk factors				
Current smoking	103/395 (26.1%)	16/74 (21.6%)	87/321 (27.1%)	P = 0.380
Hypertension	256/389 (65.8%)	55/73 (75.3%)	201/316 (63.6%)	P = 0.075
Hyperlipidemia	109/395 (27.6%)	26/74 (35.1%)	83/321 (25.9%)	P = 0.114
Diabetes	123/395 (31.1%)	29/74 (39.2%)	94/321 (29.3%)	P = 0.125
Family history of CAD	97/395 (24.6%)	19/74 (25.7%)	78/321 (24.3%)	P = 0.881
BMI >30 (kg/m^2^)	181/383 (47.3%)	29/74 (39.2%)	152/309 (49.2%)	P = 0.154
Prior coronary disease	113/389 (29.1%)	37/74 (50.0%)	76/315 (24.1%)	**P <0.0001**
Prior MI	71/389 (18.3%)	28/74 (37.8%)	43/315 (13.7%)	**P <0.0001**
Prior PCI	61/388 (15.7%)	19/74 (25.7%)	42/314 (13.4%)	**P = 0.013**
Prior CABG	35/390 (9.0%)	8/74 (10.8%)	27/316 (8.5%)	P = 0.504
Prior CHF	54/389 (13.9%)	14/73 (19.2%)	40/316 (12.7%)	P = 0.187
Prior PVD	21/395 (5.3%)	4/74 (5.4%)	17/321 (5.3%)	P = 1.000
Prior stroke	41/395 (10.4%)	7/74 (9.5%)	34/321 (10.6%)	P = 1.000

MACE—major adverse cardiac events, CAD—coronary artery disease, PVD—peripheral vascular disease, BMI—body mass index, MI—myocardial infarction, PCI—percutaneous coronary intervention, CABG coronary artery bypass grafting, CHF—congestive heart failure.

**Table 2:** Frequency of prehospital modified HEART Pathway determinants (N = 395).

Risk Stratification Measure	Number	Percent
HEAR Score		
History		
Slightly suspicious (0 points)	106	26.8%
Moderately suspicious (1 point)	146	37.0%
Highly suspicious (2 points)	143	36.2%
ECG		
Normal (0 points)	255	64.7%
Nonspecific changes (1 point)	109	27.7%
Acute ischemic changes (2 points)	30	7.6%
Age		
<45 (0 points)	63	16.0%
45–65 (1 point)	206	52.1%
>65 (2 points)	126	31.9%
Number of Risk Factors		
0 (0 points)	41	10.4%
1–2 (1 point)	142	36.0%
≥ 3 (2 points)	211	53.6%
Total HEAR Score		
0	6	1.5%
1	24	6.1%
2	36	9.2%
3	62	15.8%
4	103	26.2%
5	79	20.1%
6	61	15.5%
7	18	4.6%
8	4	1.0%
Prehospital Modified HEART Pathway (PMHP)		
High Risk	28	7.1%
Moderate Risk	243	61.5%
Low Risk	124	31.4%

ACS—acute coronary syndrome, cTn—cardiac troponin, HEAR—History, ECG, Age, Risk factors.

The PMHP identified 7.1% (28/395) as high risk, 31.4% (124/395) as low risk and 61.5% (243/395) as moderate risk. Among the 28 patients identified as high-risk, 17 had 30-day MACE, resulting in a PPV of 60.7% (95%CI 40.6–78.5%). Specificity for the detection of 30-day MACE was 96.6% (95% CI 94.0–98.3%). In the 124 low risk patients, 7 had 30-day MACE yielding a NPV of 94.4% (95%CI 88.7–97.7%). Sensitivity for the detection of 30-day MACE was 90.5% (95% CI 81.5–96.1%). Among moderate risk patients MACE occurred in 20.6% (50/243; 95%CI 15.6–26.0%).

**Fig 3:**
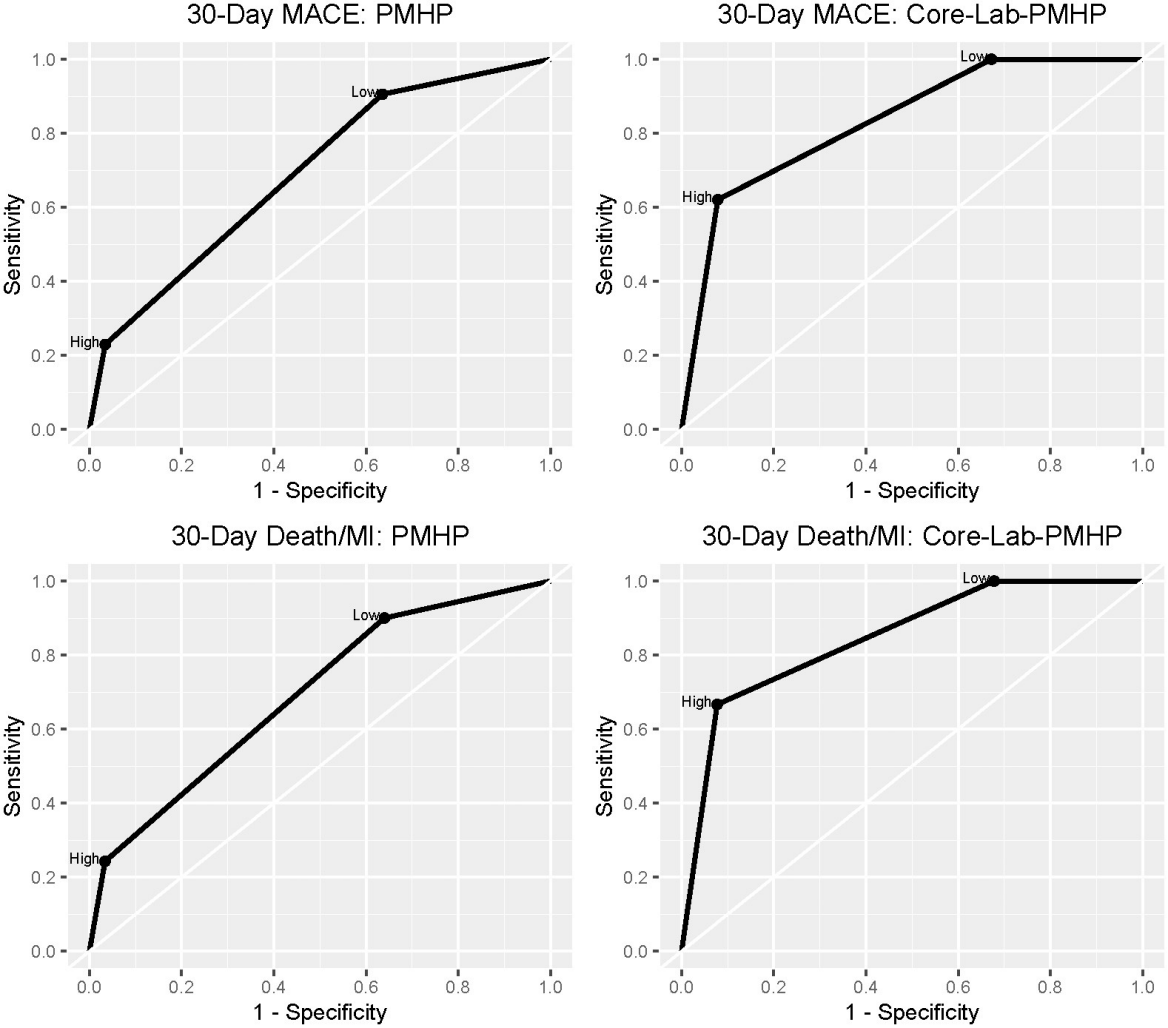
Receiving operator curves for prehospital modified HEART Pathway and core-lab-prehospital modified HEART Pathway for 30-day MACE and Death/MI.

The core-lab-PMHP yielded 100% sensitivity (95% CI: 93.8–100%) and NPV (95% CI 95.2–100%) for 30-day MACE. A summary of the prognostic characteristics for the PMHP and core-lab-PMHP assessments for the detection of MACE and Death/MI at 30 days are presented in Table 3 and receiving operator curves are presented in Fig 3. Test characteristics of the PHMP for index visit outcomes are summarized in S2 Table. The seven patients identified as low risk by paramedics who had 30-day MACE, are described in Table 4. In one of these patients a paramedic incorrectly calculated an EMS HEAR score of 2 when based on EMS chart review the correct HEAR score was 4.

**Table 3:** Diagnostic characteristics for prehospital modified HEART Pathway and core-lab-prehospital modified HEART Pathway for 30-day MACE and Death/MI.

		30-day MACE		30-day Death/MI	
		PMHP	Core-Lab-PMHP	PMHP	Core-Lab-PMHP
High Risk	Specificity (95%CI)	96.6% (94.0–98.3%)	92.1% (87.9–95.3%)	96.6% (94.0–98.3%)	92.3% (88.1–95.4%)
	PPV (95%CI)	60.7% (40.6–78.5%)	66.7% (52.5–78.9%)	60.7% (40.6–78.5%)	66.7% (52.5–78.9%)
	+LR (95%CI)	6.704 (3.279–13.705)	7.897 (4.852–12.850)	7.175 (3.517–14.640)	8.630 (5.328–13.977)
Low Risk	Sensitivity (95%CI)	90.5% (81.5–96.1%)	100% (93.8–100%)	90.0% (80.5–95.9%)	100% (93.4–100%)
	NPV (95%CI)	94.4% (88.7–97.7%)	100% (95.2–100%)	94.4% (88.7–97.7%)	100% (95.2–100%)
	-LR (95%CI)	0.260 (0.126–0.533)	0 (0-NA)	0.278 (0.136–0.569)	0 (0-NA)

PMHP—prehospital modified HEART Pathway, MACE—major adverse cardiac events, NPV—negative predictive value, PPV—positive predictive value, -LR—negative likelihood ratio, +LR positive likelihood ratio, POC—point of care.

**Table 4:** Description of low-risk patients by prehospital modified HEART Pathway who had 30-day MACE.

Age	Sex	Race/Ethnicity	Comorbidities	EMS HEAR Score (History value)	i-STAT cTn (0–0.08 ng/mL)	Initial Hospital Lab cTn ng/mL (ref range)	Peak Hospital Lab cTn ng/mL (ref range)	Event	Notes
54 yr	M	Latino	Known CAD	3 (2)	0.02	0.051 (0–0.025)	0.053 (0–0.025)	Type II NSTEMI	Cocaine abuse
43 yr	F	AA	HTN, HCL, DM, Obesity	2(0)	0.01	0.033 (0–0.025)	0.053 (0–0.025)	Type II NSTEMI	Hypoxic asthma exacerbation with demand ischemia
42 yr	M	AA	Smoker, HTN	2(1)	0.05	0.058 (0–0.040)	0.109 (0–0.040)	Type II NSTEMI	Hypertensive urgency with demand ischemia
45 yr	F	White	HTN	1(0)	0	0.044 (0–0.025)	0.045 (0–0.025)	Type II NSTEMI	Hypertension with demand ischemia
53 yr	M	AA	HTN, known CAD	3(0)	0	0.037 (0–0.025)	0.044 (0–0.025)	Type II NSTEMI	Hypertensive heart and kidney disease, demand ischemia
35 yr	M	AA	Smoker, HIV	2(1)	0	0.045 (0–0.040)	0.045 (0–0.040)	Type II NSTEMI	Methamphetamine abuse
53 yr	M	White	Smoker, DM, HTN, HCL, Obesity	3(0)	0	0.075 (0–0.040)	0.086 (0–0.040)	Type I NSTEMI	Stent placed in left circumflex for 90% stenosis

EMS—Emergency Medicine Services, POC—point-of-care, HEAR—History, ECG, Age, Risk factors, MACE—major adverse cardiac events, M—male; F—female; AA—African American; CAD—Coronary Artery Disease; HTN—Hypertension; HCL—Hypercholesterolemia; DM—Diabetes Mellitus; cath—catheterization; HIV—Human Immunodeficiency Virus Infection; NSTEMI—non-ST Elevation Myocardial Infarction; CMR—Cardiovascular Magnetic Resonance imaging.

## Discussion

This study demonstrates that a prospective application of the PMHP including a POC cTn completed by paramedics during ambulance transport achieves high specificity and NPV for 30-day MACE. A high risk PMHP assessment resulted in a specificity of 96.6% and PPV of 60.7% for 30-day MACE. Meanwhile, a low risk PMHP assessment was associated with a NPV of 94.4% and a sensitivity of 90.5% for 30-day MACE. Thus, this study offers proof of concept that paramedics are able to accurately risk stratify patients with possible ACS, beyond STEMI recognition, by using a PMHP with POC cTn.

While few patients with a low risk prognostic PMHP assessment had short-term MACE events, its sensitivity and NPV for 30-day MACE was insufficient to exclude MACE. However, a 90.5% sensitivity and 94.4% NPV may be adequate for the purpose of triaging patients to a referral hospital based on a low-risk assessment. Furthermore, our data from the core-lab-PMHP suggests that as newer, more sensitive, POC cTn assays become available in the US, the sensitivity of the PMHP will improve [27, 28]. In fact, in this cohort the core-lab-PMHP (the combination of a HEAR score with a core lab cTn measure from prehospital blood) achieved 100% sensitivity and NPV for 30-day MACE.

These data suggest that in the future, mobile integrated healthcare models which incorporate EMS risk stratification algorithms could allow patients who are very-low-risk of MACE to avoid transport to a hospital ED. With advances in POC cTn and well-validated algorithms a subset of patients with acute chest pain could be effectively ruled-out for adverse cardiac events by an on-scene paramedic and a telehealth provider. Though other emergent etiologies of chest pain must also be considered and ruled out, these patients could theoretically be scheduled for a rapid outpatient follow-up appointment avoiding ambulance transport and ED evaluation if they were very low risk for any dangerous etiology of chest pain [29]. The Emergency Triage, Treat and Transport (ET3) Model, a voluntary payment model recently announced by CMS, allows for payment to EMS agencies that transport patients to destinations other than the ED or use telehealth following a 911 call. This policy change may enable EMS agencies to execute more efficient EMS chest pain risk stratification protocols once they are further validated.

A high-risk PMHP assessment was highly specific for 30-day MACE, with a moderate PPV and positive likelihood ratio. This suggests that an elevated POC cTn in the prehospital setting indicates a high likelihood that the patient will be diagnosed with an adverse cardiac event within the next thirty days. Thus, our results support the concept of using POC cTn in the prehospital setting for the early identification of non-STEMI ACS. Patients with elevated POC cTn measures could be treated more aggressively and triaged to facilities with on-site cardiac catheterization laboratories. This practice could avoid costly and inefficient downstream inter-facility transfers and may improve patient outcomes. Conversely, patients without an elevated prehospital POC cTn measure, who are at moderate or low risk by the PMHP, could be safely cared for at local community hospitals that do not have interventional cardiology services.

This study has several limitations. Patients were included from three EMS agencies and were transported to a single academic medical center. Although we suspect there are many similarities between our EMS agencies, medical center, and patients to those across the US, our results may not be generalizable to all agencies, centers, and patients. In addition, because our cohort was accrued by treating paramedics as a convenience sample, this study is limited by selection bias. Although our 30-day MACE rate of 18.7% is higher than most ED cohorts, it is similar to other studies focused on EMS chest pain care [5, 17, 30]. The time of patient’s chest pain onset relative to calling 911 and paramedic arrival was not collected. This prevented the ability to differentiate early presenters from late presenters. Previous studies have demonstrated that cTn measurement is less sensitive for the detection of MI among early presenters compared to late presenters [31, 32]. Thus, a single negative POC troponin among early presenter is likely of little value. Conversely a single troponin in a patient who has prolonged constant pain for greater than eight hours may be sufficient and is consistent with published guidelines [33]. Thus, the proportion of both early are late presenters in this cohort may have impacted PMHP assessments. However, when EMS blood was tested using the more sensitive core lab assay and combined with a prehospital HEAR score (the core-lab-PMHP) the sensitivity and NPV for MACE reached 100%, regardless of time of onset.

This study suggests that a PMHP has prognostic value for 30-day MACE. The specificity of a high-risk PMHP assessment for 30-day MACE was high. While further validation is needed, these results suggest that a structured prehospital risk assessment with POC cTn could be used to facilitate early identification of high-risk patients who may benefit from rapid treatment and triage to tertiary care facilities with interventional cardiac catheterization laboratory capability. Furthermore, the NPV and sensitivity of the PMHP for 30-day MACE may be sufficient to enable the triage of low-risk patients to referral hospitals without interventional cardiology capabilities. In addition, our data suggest that sensitivity and NPV of the PMHP will improve as next-generation POC cTn assays become available. A larger-scale multisite validation of the PMHP is needed to determine whether broad prehospital implementation is indicated.

## Supporting information

S1 File (PDF)

S2 File TREND statement checklist. (PDF)

S3 File CONSORT 2010 flow diagram. (DOC)

S4 File (DOCX)

S1 Table Patient characteristics for the entire cohort, the 395 patients in the analysis set with completed prehospital modified HEART Pathway (PMHP) assessments, and the 111 patients without completed PMHP assessments. (DOCX)

S2 Table Diagnostic characteristics for prehospital modified HEART Pathway and core-lab-prehospital modified HEART Pathway for index MACE and Death/MI. (DOCX)
