# Supplementary material for: Prehospital use of a modified HEART Pathway and point-of-care troponin to predict cardiovascular events
Source: PLoS One. 2020 Oct 7;15(10):e0239460. doi: 10.1371/journal.pone.0239460 (PMC7540888; doi:10.1371/journal.pone.0239460)
Supplement: S1 File — (PDF) [file pone.0239460.s001.pdf]

Check for updates

Point-of-Care Troponin Testing during Ambulance Transport to Detect Acute Myocardial Infarction

Jason P. Stopyra, MD, MS, FACEP, FAEMS, Anna C. Snavely, PhD, James F. Scheidler, MD, Lane M. Smith, MD, PhD, Robert D. Nelson, MD, James E. Winslow, MD, Gregory J. Pomper, MD, Nicklaus P. Ashburn, MD, Nella W. Hendley, MS, Robert F. Riley, MD, MS, Lauren E. Koehler, EMT-P, Chadwick D. Miller, MD, MS, Simon A. Mahler, MD, MS

Abstract

Objective: Use of point-of-care (POC) troponin (cTn) testing in the Emergency Department (ED) is well established. However, data examining POC cTn measurement in the prehospital setting, during ambulance transport, are limited. The objective of this study was to prospectively test the performance of POC cTn measurement by paramedics to detect myocardial infarction (MI) among patients transported to the ED for acute chest pain. **Methods:** A prospective cohort study of adults with non-traumatic chest pain was conducted in three Emergency Medical Services agencies (December 2016 to January 2018). Patients with ST-elevation MI on ECG were excluded. During ambulance transport paramedics initiated intravenous access, collected blood, and used a POC device (i-STAT; Abbott Laboratories) to measure cTn. Following ED arrival, participants received standard evaluations including clinical blood draws for cTn measurement in the hospital central lab (AccuTnI +3 assay; Beckman Coulter, or cTnI-Ultra assay; Siemens). Blood collected during ambulance transport was also analyzed for cTn in the central lab. Index visit MI was adjudicated by 3 experts using central lab cTn measures from the patient's clinical blood draws. Test characteristics (sensitivity, specificity, and predictive values) for detection of MI were calculated for POC and central lab cTn measurement of prehospital blood and compared with McNemar's test. **Results:** During the study period prehospital POC cTn results were obtained on 421 patients, of which 5.0% (21/421) had results >99th percentile upper reference limit. MI was adjudicated in 16.2% (68/421) during the index visit. The specificity and positive predictive value of the POC cTn measurement were 99.2% (95% CI 97.5–99.8%) and 85.7% (95% CI 63.7–97.0%) for MI. However, the sensitivity and NPV of prehospital POC cTn were 26.5% (95% CI 16.5–38.6%) and 87.5% (95% CI 83.9–90.6%). Compared to POC cTn, the central lab cTn measurement of prehospital blood resulted in a higher sensitivity of 67.9% (95% CI 53.7–80.1%, $p < 0.0001$), but lower specificity of 92.4% (95% CI 88.4–95.4%, $p = 0.0001$). **Conclusions:** Prehospital POC i-STAT cTn measurement in patients transported with acute chest pain was highly specific for MI but had low sensitivity. This suggests that prehospital i-STAT POC cTn could be useful to rule-in MI, but should not be used to exclude MI. **Key words:** chest pain; troponin; point-of-care; prehospital; myocardial infarction

PREHOSPITAL EMERGENCY CARE 2020;00:000–000

Received October 16, 2019 from WFUSOM Emergency Medicine, Winston-Salem, North Carolina (JPS, JFS, LMS, RDN, JEW, NPA, NWH, LEK, CDM, SAM); WFUSOM Biostatistics and Data Science, Winston-Salem, North Carolina (ACS); WFUSOM Pathology, Winston-Salem, North Carolina (GJP); The Christ Hospital Heart and Vascular Center Cardiology, Cincinnati, Ohio (RFR). Revision received January 15, 2020; accepted for publication January 21, 2020.

Presentations: SAEM 2019 Annual Meeting, Las Vegas, NV May 2019.

This study was funded by Abbott Point of Care. Dr. Stopyra also receives research funding from Roche Diagnostics. Dr. Mahler also receives research funding from Donaghue Foundation and the Association of American Medical Colleges (AAMC), Roche Diagnostics, Siemens, Ortho Clinical Diagnostics, Creavo Medical Technologies, PCORI, AHRQ, and NHLBI (1 R01 HL118263-01). Dr. Miller also receives research funding/support from Siemens, Abbott Point of Care, Creavo Medical Technologies, and 1 R01 HL118263. Dr. Mahler is the Chief Medical Officer for Impathiq Inc. Dr. Snavely also receives research funding/support from NHLBI (1 R01 HL118263).

The opinions, findings, and conclusions or recommendations expressed in this publication are those of the authors and do not necessarily reflect the views of Abbott Point of Care. The funding agency did not participate in or direct study design, data analysis, or manuscript preparation. We would like to acknowledge and thank Roy Alson, Danielle Black, Maggie Bohannon, Blake Briggs, David Conner, Louis Fornage, Jeremiah Gaddy, Rachel Gillespie, Angelica Mack, Kayla Mayes, James McAlister, Evan McMurray, Adam Pflum, Greg Russell, Tyler Woodrum, David Zhao and especially all of the EMS providers for the assistance they provided on this project.

Address correspondence to Jason P. Stopyra, MD, MS, FACEP, FAEMS, Department of Emergency Medicine, Wake Forest School of Medicine (WFSOM), Medical Center Boulevard, Winston-Salem, NC 27157, USA. E-mail: jstopyra@wakehealth.edu

© 2020 National Association of EMS Physicians

doi:10.1080/10903127.2020.1721740

Introduction

Patients experiencing chest pain commonly call 911 for assistance due to fear they are having a heart attack (1). In fact, many patients that seek care in the Emergency Department (ED) for symptoms

PROOF ONLY

concerning for acute coronary syndrome arrive by ambulance (2). Despite frequent chest pain encounters, current Emergency Medical Services (EMS) risk stratification protocols are limited to electrocardiogram (ECG) screening for an ST-segment elevation myocardial Infarction (STEMI). Thus in the prehospital setting, patients at high risk for non-ST-segment elevation myocardial infarction (NSTEMI) are not differentiated from patients with benign chest pain etiologies. Furthermore, patients with NSTEMI may be transported to hospitals, which lack interventional coronary catheterization capabilities, leading to eventual transfer to another facility, which inefficiently uses limited EMS resources, increases cost, and delays definitive care.

Point-of-care (POC) troponin (cTn) measurement can rapidly identify patients with NSTEMI and is well validated in the ED setting (3, 4). However, POC cTn testing has yet to be extended to the prehospital setting. Thus far, the data on conducting POC cTn measurement in the prehospital setting is very limited (5–9). The accuracy and feasibility of POC cTn measurement in the back of a moving ambulance was tested on an Abbott POC device on "simulated runs" (9). This study found that there was no significant difference in results between assays performed in the moving ambulance and those performed in the ED. A Danish study, which enrolled 985 prehospital patients and tested POC cTn (Roche Diagnostics, GmbH, Mannheim, Germany) demonstrated a sensitivity for myocardial infarction (MI) of 39% (95% CI 32–46%) and a specificity of 95% (95% CI 94–97%) (10). These test characteristics fell below the acceptable level to be acted upon clinically. Two studies of prehospital POC cTn testing in Canada and Europe resulted in reduced ED length of stay and demonstrated moderate accuracy for predicting Major Adverse Cardiac Events at 45 days, respectively (11, 12). However, the prospective use of POC cTn by paramedics on patients with acute chest pain during prehospital transport has yet to be tested in the United States.

The primary objective of this study was to establish the diagnostic performance of a prehospital POC cTn assay (Abbott's i-STAT®) performed by a paramedic prior to ED arrival. In addition, we compared POC cTn measurements to measurements in a hospital laboratory. Finally, the performance of POC cTn cut points, at the upper reference limit (URL) and limit of detection (LOD), was compared to determine the optimal cTn threshold for a prehospital chest pain patient population.

METHODS

Study Design

We conducted a prospective cohort study at three EMS systems from December 2016 to January 2018. One hundred fifty paramedics at participating agencies were trained to use the i-STAT device (Abbott Laboratories, Chicago, IL) for POC cTn measurement. In this study, paramedic POC cTn measurement results were not used clinically to alter treatment or destination protocols. Study blood collection was within the scope of practice of paramedics, who routinely perform venipuncture and conduct POC testing (e.g., blood glucose) on acutely ill patients. Thus, this study was performed under a waiver of informed consent obtained from the Wake Forest University Health Sciences Institutional Review Board. We conducted this study in compliance with the STARD guidelines and registered with clinicaltrials.gov (NCT02709135) prior to patient accrual.

Study Setting

Three county EMS agencies located in the piedmont region of North Carolina participated in this study. Stokes County EMS is a rural agency that has 34 medics, 5 ambulances, and completes 6000 transports each year. Surry County EMS is also a rural agency with 73 medics, 7 ambulances, and completes approximately 17,000 transports annually. Forsyth County EMS, an urban agency, has approximately 80 medics, 16 ambulances in service, and completes about 35,000 patient transports annually. Medics were required to include only patients transported to Wake Forest Baptist Health (WFBH) ED. WFBH is a tertiary care, level 1 trauma and burn center for adults and pediatrics with 821 licensed beds, full specialty/subspecialty availability, and around the clock advanced cardiac catheterization capability. The ED has 47 beds with an annual volume of approximately 105,000 visits. The ED is staffed by Board Certified or Board Eligible Emergency Physicians 24 h per day, 7 days a week who directly provide and oversee care provided by residents and advanced practitioners.

Participants

A convenience sample of adult patients ≥21 years old with acute, non-traumatic chest pain, without evidence of STEMI on ECG (ST-segment elevation in contiguous leads on any ECG (≥1 mV)) transported to WFBH ED was accrued. Patients were excluded if they were being transferred from other

PROOF ONLY

TABLE 1. Patient characteristics among patients with and without MI

Patient characteristic	Total, $N = 421$	Patients with MI, $n = 68$	Patients without MI, $n = 353$	MI vs. no MI
Age years, mean ± SD	58.8 ± 15.2	64.4 ± 13.3	57.7 ± 15.3	**$p < 0.001$**
Sex (female)	224 (53.2%)	30 (44.1%)	194 (55.0%)	$p = 0.112$
Race				
Caucasian	217 (51.9%)	34 (50.8%)	183 (52%.1)	$p = 0.073$
African American	178 (42.6%)	27 (40.3%)	151 (43.0%)	
Asian	4 (1.0%)	3 (4.5%)	1 (0.3%)	
Native American	1 (0.2%)	0 (0%)	1 (0.3%)	
Other	18 (4.3%)	3 (4.5%)	15 (4.3%)	
Ethnicity (Hispanic)	18 (4.3%)	2 (2.9%)	16 (4.5%)	$p = 0.750$
Risk factors				
Current smoking	105 (24.9%)	15 (22.1%)	90 (25.5%)	$p = 0.647$
Hypertension	275 (66.3%)	51 (76.1%)	224 (64.4%)	$p = 0.068$
Hyperlipidemia	112 (26.6%)	22 (32.4%)	90 (25.5%)	$p = 0.294$
Diabetes	129 (30.6%)	24 (35.3%)	105 (29.8%)	$p = 0.390$
Family history of CAD	104 (24.7%)	16 (23.5%)	88 (24.9%)	$p = 0.879$
BMI >30 (kg/m^2)	232 (47.2%)	29 (42.7%)	170 (50.0%)	$p = 0.290$
Prior coronary disease	121 (29.2%)	32 47.1%)	89 (25.7%)	**$p < 0.001$**
Prior MI	78 (18.8%)	25 (36.8%)	53 (15.3%)	**$p < 0.001$**
Prior PCI	65 (15.7%)	16 (23.5%)	49 (14.1%)	$p = 0.067$
Prior CABG	43 (10.0%)	9 (13.2%)	34 (9.8%)	$p = 0.386$
Prior CHF	56 (13.5%)	16 (23.9%)	40 (11.5%)	**$p = 0.011$**
Prior PVD	20 (4.8%)	3 (4.4%)	17 (4.8%)	$p = 1.000$
Prior stroke	42 (10.0%)	7 (10.3%)	35 (9.9%)	$p = 1.000$

MI, myocardial infarction; SD, standard deviation; CAD, coronary artery disease; BMI, body mass index; PCI, percutaneous coronary intervention; CABG coronary artery bypass grafting; CHF, congestive heart failure; PVD, peripheral vascular disease.

PROOF ONLY

acute care facilities or first medical contact to ED arrival time was anticipated to be less than 5 min. In addition, patients were also excluded if they had concomitant non-cardiac medical, surgical, or psychiatric emergencies, were receiving hospice care, or had unstable vital signs as defined as symptomatic hypotension (systolic blood pressure < 90 mm Hg), tachycardia (heart rate >120), bradycardia (heart rate <40), and hypoxemia (<90% pulse-oximetry on room air or normal home oxygen flow rate).

Study Protocol

Training was provided to paramedics to identify subjects appropriate for inclusion through in-person teaching and self-learning video refresher modules. Abbott i-STAT device training was conducted by Abbott Point of Care trainers. Paramedics were taught proper storage, device maintenance, quality assurance procedures, and result interpretation and reporting.

Eighteen ambulances across Forsyth, Surry, and Stokes Counties were equipped with i-STAT devices to enroll participants following training. The i-STAT devices were attached to the paramedic workstation in the back of the ambulance using touch fastener strips (Velcro Industries, United Kingdom). Routine protocol-driven chest pain care, triage and destination plans for these EMS counties include obtaining intravenous access (IV), ECG, and the administration of aspirin, nitroglycerin, and supplemental oxygen as needed. Blood was collected from eligible patients in blood tubes through the intravenous catheter that was just inserted. The i-STAT cartridge was then filled with a sample of this blood using a syringe or a blood transfer device (i-STAT dispensing tip) and then placed in the analyzer. Upon arrival at the ED the tube of blood drawn by EMS was sent to the hospital central lab for cTn testing. The i-STAT analyzer was brought to the docking station to allow for data to be downloaded. Error codes from i-STAT use were recorded and included analyzer error (dead battery, temperature out of range, motor moved too far, and electronic failure), structural cartridge errors (cartridge preburst, amperometric sensor problem, poor cartridge contact, quality control failure, poor electrical connection, analysis fluid mixed with sample and atypical data stream), movement of analysis fluid during the cartridge run, poor filling errors and other errors (no error code recorded).

During study training it was emphasized that the i-STAT troponin results were not to influence the patient's transportation destination or care. A cTn cut point of 0.080 ng/ml corresponding to the published 99th percentile URL for the i-STAT assay was used for a positive result. The POC cTn results were shared with the ED care team as part of their typical transfer of care report. A study specific form was used to assist with this transfer of information, which clearly stated that the POC cTn value was for

research purposes only and was not to be used to alter a patient's clinical care. ED nurses and providers were trained to continue to use central-lab troponin for confirmation.

Quality control measures were put in place for the i-STAT devices, cTnI cartridges, and paramedics. Temperature of both the i-STAT device and cartridges were closely monitored to ensure they stayed within the manufacturer's specified range. On the ambulances, i-STAT devices and the cTn cartridges were stored in a temperature monitored cooler (Koolatron, Batavia, NY), that was capable of providing both heating and cooling to maintain a constant temperature of 22 degrees Celsius. Coolers were plugged into an electrical source on the ambulance and operated continuously. Unused EMS cartridges were replaced every two weeks from a stock stored in a 6-degree Celsius refrigerator. Each batch of new cartridges was subject to a quality control test prior to use per manufacturer specifications. The i-STAT software was updated every 6 months per manufacturer specifications and the machine was required to pass a simulation prior to use. Paramedics were also required to complete a 6-month competency testing through the completion of two successful quality control tests.

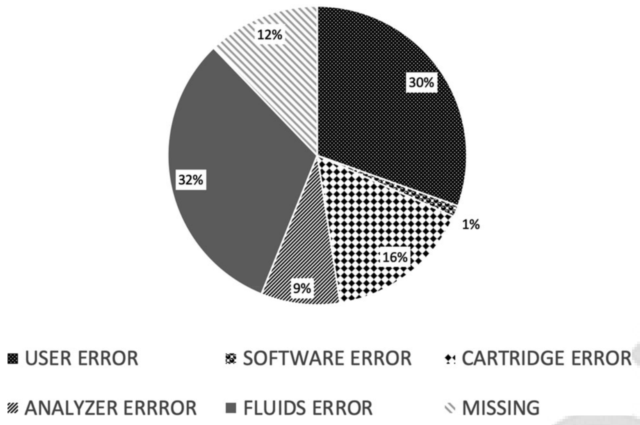

FIGURE 1. i-STAT error codes. User error: codes 1, 123, 128, 132, 133, and 134; software error: code 13; cartridge error: codes 21, 43, 126, 127, 129, and 149; analyzer error: codes 50, 66, and 79; fluids error: codes 136, 145, and 146

Upon arrival in the WFBH ED, participants received standard ED chest pain evaluation, which included an ECG and blood draws for cTn measurement. Blood was obtained in the ED at presentation and 3 h following presentation and used for cTn measurement using the central lab [AccuTnI + 3 assay (Beckman Coulter, CA) with a 0.025 ng/ml URL or TnI-Ultra assay (Siemens, Munich Germany) with a 0.040 ng/ml URL] as part of normal care. Blood samples collected at 0 and 3 h were also tested using the Abbott i-STAT POC assay in the WFBH blood gas laboratory by trained laboratory personnel.

Outcomes. The primary objective of this project was to analyze the performance of a prehospital POC cTn assay (Abbott's i-STAT®), performed by a paramedic prior to ED arrival, to detect index visit MI. Patients were followed through their index visit by electronic medical record surveillance to identify MI. MI was defined using the Joint European Society of Cardiology/American College of Cardiology Foundation/American Heart Association/World

COLOR Online / B&W in Print

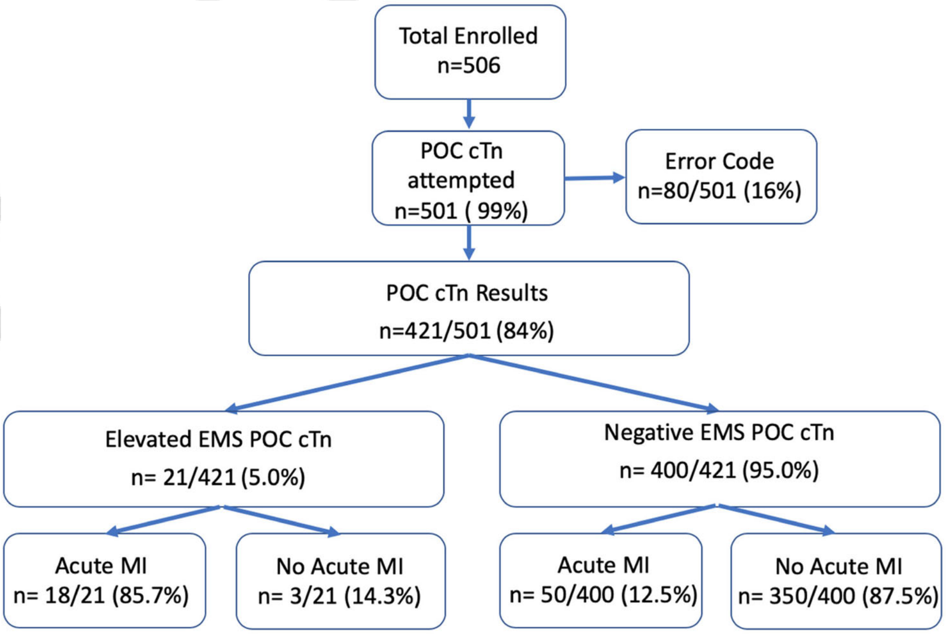

FIGURE 2. Study flow diagram. POC, point-of-care; cTn, cardiac troponin; EMS, emergency medical services; MI, myocardial infarction.

TABLE 2. Test characteristics for cTn assay detection of index visit MI at each blood draw

Blood draw	EMS		ED arrival		3-h	
Assay	EMS i-STAT	Central	Lab i-STAT	Central	Lab i-STAT	Central
Sensitivity (95% CI)	26.5% (16.5–38.6%)	67.9% (53.7–80.1%)	34.6% (22.0–49.1%)	77.9% (66.2–87.1%)	37.9% (20.7–57.7%)	85.1% (74.3–92.6%)
Specificity (95% CI)	99.2% (97.5–99.8%)	92.4% (88.4–95.4%)	99.6% (97.5–100.0%)	95.8% (93.0–97.8%)	98.8% (93.4–100%)	96.0% (92.9–98.0%)
NPV (95% CI)	87.5% (83.9–90.6%)	93.2% (89.3–96.0%)	86.8% (82.0–90.7%)	95.2% (92.2–97.3%)	81.8% (72.8–88.9%)	96.3% (93.4–98.2%)
PPV (95% CI)	85.7% (63.7–97.0%)	65.5% (51.4–77.8%)	94.7% (74.0–99.9%)	80.3% (68.7–89.1%)	91.7% (61.5–99.8%)	83.8% (72.9–91.6%)
+LR (95% CI)	31.15 (9.43–102.83)	8.97 (5.61–15.67)	77.54 (10.59–567.77)	18.71 (10.83–32.32)	31.10 (4.20–230.48)	21.19 (11.77–38.14)
–LR (95% CI)	0.74 (0.64–0.86)	0.35 (0.24–0.51)	0.66 (0.54–0.80)	0.23 (0.15–0.36)	0.63 (0.47–0.84)	0.16 (0.09–0.28)

cTn, cardiac troponin; MI, myocardial infarction; EMS, Emergency Medical Services; ED, Emergency Department; CI, confidence interval; NPV, negative predictive value; PPV, positive predictive value; +LR, positive likelihood ratio; –LR, negative likelihood ratio.

Health Federation Task Force universal definition (13). MI events were adjudicated by three cardiovascular experts (two primary reviewers and one secondary reviewer). Adjudicators were provided access to all clinical data from the patient's index visit, including cTn measures from the core-lab, but were blinded to EMS POC cTn results. Any discrepancies among the two primary cardiologist reviewers were resolved by an emergency physician that served as the third reviewer.

Data Analysis

Test characteristics, including sensitivity, specificity, positive and negative predictive values (PPV and NPV), and positive and negative likelihood ratios (+LR and –LR) for index visit MI were calculated for EMS blood, ED arrival blood, and 3-h blood run with i-STAT and central lab assays. Corresponding exact binomial 95% confidence intervals were computed for sensitivity, specificity, PPV and NPV. For the positive and negative likelihood ratios, 95% confidence intervals were calculated using the method of Simel et al. (14). Patient characteristics were summarized and compared between those with and without MI using t-tests or Fisher's exact tests. Kappa and raw agreement analyses were performed between i-STAT POC and Central Lab analyses for EMS sample, ED arrival blood and 3-h clinical draw. Acceptable agreement for kappa assessments was defined *a priori* as $\geq$0.60. Sensitivity and specificity were compared between tests using the same blood using exact McNemar's tests. The primary analysis of the performance of the i-STAT POC troponin was tested using a cut point of 0.080 ng/mL, which corresponds to the package insert 99th percentile URL for the assay. A secondary analysis was conducted using the LOD as the cut point and a Receiver Operating Characteristic (ROC) plot was generated to visualize other potential cut points. Statistical analysis was performed using SAS 9.4 (SAS Institute, Cary, NC) and R 3.5.1 (www.R-project.org).

RESULTS

Ninety-seven paramedics from three EMS agencies accrued 506 eligible patients from December 2016 to April 2018. Prehospital POC cTn measurement was attempted in 99% (501/506) of patients, with 83.2% (421/506) receiving numeric results and 15.8% (80/506) resulting in error codes. MI was present in 16.2% (68/421) during the index visit. Patient characteristics in the cohort and among those with and without MI are described in Table 1. The

PROOF ONLY

TABLE 3. Agreement between i-STAT and central lab cTn assays

Troponin comparison	Raw agreement	Kappa (95% CI)
Prehospital blood draw on EMS i-STAT vs. central lab assay	85.9% (95% CI 81.4–89.6%)	0.34 (CI: 0.20–0.48)
ED arrival draw, blood gas lab i-STAT vs. central lab assay	89.4% (95% CI 85.1–92.9%)	0.53 (CI: 0.38–0.67)
3-h clinical draw blood gas lab i-STAT vs. central lab assay	85.3% (95% CI 77.3–91.4%)	0.50 (CI:0.31–0.70)

cTn, cardiac troponin; CI, confidence interval; EMS, Emergency Medical Services; ED, Emergency Department.

breakdown of i-STAT device errors that occurred during this study is summarized in Figure 1.

The study flow diagram is presented in Figure 2. Among the 421 patients with an EMS POC cTn result (without an error code), the specificity of the EMS i-STAT POC cTn result was 99.2% (95% CI 97.5–99.8%) while the specificity of the central cTn result was 92.4% (95% CI 88.4–95.4%) on the same blood ($p = 0.0001$). The sensitivity of the i-STAT cTn measure for MI was 26.5% (95% CI 16.5–38.5%) compared to a sensitivity of 67.9% (95% CI 53.7–80.1%) for central lab cTn analysis of the same blood sample ($p < 0.0001$). A complete comparison of the test characteristics for cTn assay detection of MI using the i-STAT and central laboratory assays at the prehospital (EMS), first (ED arrival) and 3-h clinical blood draws is presented in Table 2.

Agreement (on elevated vs. negative results) and Kappa between i-STAT POC cTn measurement and central lab assay for each time point is presented in Table 3. Each Kappa was below 0.60, indicating agreement was not "acceptable." Most disagreements occurred in patients with elevated central lab troponins and negative POC results (raw disagreement 14.1%, 95% CI 10.4–18.6%). For the blood draw on ED arrival, all disagreements occurred in patients with elevated central lab troponins and negative POC results (raw disagreement 10.5%, 95% CI 7.1–14.9%). For the 3-h blood sample most disagreements occurred in patients with elevated central lab troponins and negative POC results (raw disagreement 14.7%, 95% CI 8.6–22.7%).

The LOD of the i-STAT for this cohort was 0.010 ng/mL. The use of this value as the cut point for EMS blood cTn testing improved sensitivity for the detection of MI from 26.5% to 79.4% ($p < 0.0001$) while decreasing the specificity from 99.2% to 74.2% ($p < 0.0001$). Test characteristics for the i-STAT using the LOD are summarized in Table 4. A ROC curve for the prediction of index MI using the EMS i-STAT cTn yielded an area under the curve of 0.823 (95% CI: 0.764–0.882); see Figure 3.

TABLE 4. Test characteristics for detection of MI using the i-STAT cTn on EMS blood with the URL vs. LOD as cut points

	EMS POC cTn cut point	
	0.08 ng/mL	LOD (0.01 ng/mL)
Sensitivity (95% CI)	26.5% (16.5–38.6%)	79.4% (67.9–88.3%)
Specificity (95% CI)	99.2% (97.5–99.8%)	74.2% (69.3–78.7%)
NPV (95% CI)	87.5% (83.9–90.6%)	94.9% (91.6–97.2%)
PPV (95% CI)	85.7% (63.7–97.0%)	37.2% (29.4–45.7%)
+LR (95% CI)	31.15 (9.43–102.83)	3.08 (2.49 – 3.82)
–LR (95% CI)	0.74 (0.64–0.86)	0.28 (0.17–0.44)

MI, myocardial infarction; cTn, cardiac troponin; URL, upper reference limit; LOD, limit of detection; EMS, Emergency Medical Services; POC, point of care; CI, confidence interval; NPV, negative predictive value; PPV, positive predictive value; +LR, positive likelihood ratio; –LR, negative likelihood ratio.

DISCUSSION

Prehospital measurement of POC cTn by paramedics during ambulance transport using the Abbott i-STAT was highly specific for MI. High specificity indicates that POC cTn can be used to rapidly rule-in NSTEMI in the prehospital setting. Thus, placing the POC cTn i-STAT device in the hands of first responders has the potential to identify patients with NSTEMI earlier and speed the delivery of potentially life-saving care. These results suggest that a POC cTn assessment integrated into EMS chest pain treatment, triage, and transportation destination plans could help ensure patients who rule-in for NSTEMI are transported directly to a hospital with cardiac catheterization capabilities. This could be particularly useful for EMS agencies in rural communities to avoid delays in definitive patient care and reduce costs associated with inter-facility transfers to PCI centers.

While the specificity and PPV of the i-STAT for detection of MI was high, sensitivity and NPV were low. The sensitivity and NPV of the POC i-STAT cTn were significantly lower than that of the central lab measurement. Only 5.0% of pre-hospital POC cTn values were elevated (> 0.08 ng/dl), despite adjudication of MI in 16.2% of this same population. Unfortunately, these results preclude the current use of the i-STAT cTn assay to safely exclude NSTEMI in the prehospital environment. Use of LOD as a cut point for POC cTn instead of the URL did substantively improve sensitivity and NPV. In addition, based on ROC analysis, the LOD is the optimal cut point for index MI. However, even at the LOD the sensitivity for detection of MI is too low to be used

PROOF ONLY

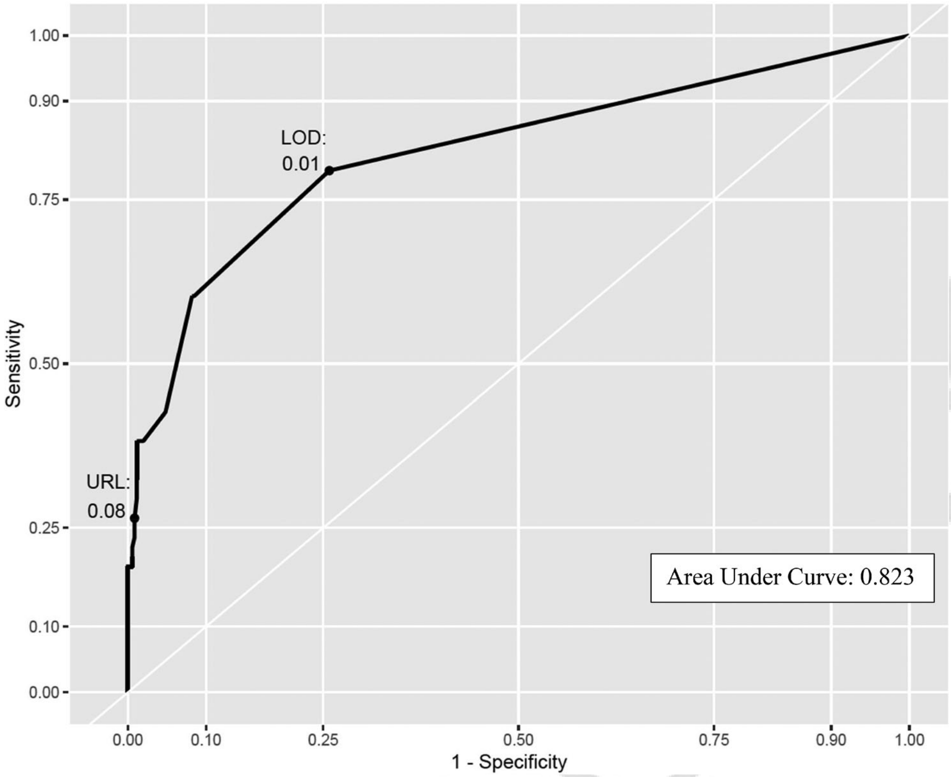

FIGURE 3. Receiver operating characteristic curve for prediction of index MI using EMS i-STAT cTn. MI – myocardial infarction; EMS, Emergency Medical Services; cTn, cardiac troponin.

clinically as a method of excluding NSTEMI in the prehospital setting. Our findings are consistent with a 2013 systematic review that evaluated POC tests in suspected MI which showed poor performance of POC testing for MI diagnosis (15, 16). However, several international diagnostics companies are working to improve the sensitivity of their POC cTn assays and make them available in the US. Recent studies have demonstrated that next generation POC devices may achieve similar performance to central lab high-sensitivity cTn assays (3, 17).

Agreement between the i-STAT measures and central lab cTn was unacceptable at each time point and fell at or below results published in prior studies (10, 18, 19). This is likely due to the lower analytical sensitivity of the POC i-STAT assay. However, since the prehospital blood draw had the lowest agreement, it is possible that paramedics' lack of experience with the i-STAT device and challenges in the back of the ambulance may have had some adverse effects on troponin results. It must also be considered that the time to chest pain onset may also be relevant factor that decreases the assay's performance.

Error code analysis of the Abbott i-STAT POC device suggests that more training is needed. Improper filling was the most common error and likely due to a lack of experience with the device and other factors related to using the device in the back of a moving ambulance. Movement and vibration errors were much less common than expected, but they were also likely mitigated by attaching the POC device to the ambulance work station with touch fastener strips.

LIMITATIONS

We conducted this study at three EMS agencies and a single academic medical center. Thus, our results may not be generalizable to all agencies, centers, and patients. In addition, because our cohort was accrued as a convenience sample, this study is limited by selection bias. Although our MI rate of 16.2% is higher than most ED cohorts, it is similar to other studies focused on EMS chest pain care (8, 20, 21). Data regarding the time of patient's chest pain onset relative to calling 911 and paramedic arrival was not collected. This made it impossible to differentiate early presenters from late presenters. Previous studies have demonstrated that cTn measurement is less sensitive for the detection of MI among early presenters compared to late presenters

(22, 23), thus the proportion of early presenters in this cohort may have impacted POC cTn results. Patient history of renal impairment was not a data point that was collected. Not all patients had their blood tested by POC and central lab devices at all time points. Finally, the i-STAT POC device had a 16% error rate in this study. This was likely due to multifactorial conditions present in a moving ambulance, which is an off-label use of the device. It should be noted that the manufacturer (Abbott Point of Care) has not validated the i-STAT cTnI test performance for use in a moving vehicle. The i-STAT instructions for use recommend that the analyzer to be placed on a flat, level, vibration-free surface for testing. Motion of the analyzer during testing is known to increase the frequency of suppressed results and quality check failures. As performing i-STAT cTnI in a moving ambulance is an off-label use it is difficult to ascertain which of the error codes reported were due to the device being used in this setting.

CONCLUSIONS

POC cTn measurement in the prehospital environment using the Abbott i-STAT device in patients transported with acute chest pain was highly specific for MI. This suggests that prehospital i-STAT POC cTn could be useful to rule-in NSTEMI and be incorporated into triage and destination plans. Patients with an elevated POC cTn measure could be triaged to a hospital with cardiac catheterization capabilities. This may speed the delivery of definitive care and reduce costs associated with interfacility transfers. However, the prehospital i-STAT cTn measure lacked sensitivity and should not be used to exclude MI. As newer POC assays with improved analytical sensitivity become available, further research in the prehospital setting will be warranted.

ORCID

Nicklaus P. Ashburn http://orcid.org/0000-0002-3466-8813

References

1. Erhardt L, Herlitz J, Bossaert L, Halinen M, Keltai M, Koster R, Marcassa C, Quinn T, van Weert H. Task force on the management of chest pain. Eur Heart J. 2002;23:1153–1176. doi:10.1053/euhj.2002.3194.
2. Kerr D, Holden D, Smith J, Kelly AM, Bunker S. Predictors of ambulance use in patients with acute myocardial infarction in Australia. Emerg Med J. 2006;23:948–952. doi:10.1136/emj.2006.038414.
3. Pickering JW, Young JM, George PM, Watson AS, Aldous SJ, Troughton RW, Pemberton CJ, Richards AM, Cullen LA, Than MP. Validity of a novel point-of-care troponin assay for single-test rule-out of acute myocardial infarction. JAMA Cardiol. 2018;3:1108–1112. doi:10.1001/jamacardio.2018.3368.
4. Body R, Almashali M, Morris N, Moss P, Jarman H, Appelboam A, et al. Diagnostic accuracy of the T-MACS decision aid with a contemporary point-of-care troponin assay. Heart. 2019;105:768–774. doi:10.1136/heartjnl-2018-313825.
5. Newman J, Aulick N, Cheng T, Faynor S, Curtis R, Mercer D, Williams J, Hobbs G. Prehospital identification of acute coronary ischemia using a troponin T rapid assay. Prehosp Emerg Care. 1999;3:97–101. doi:10.1080/10903129908958914.
6. Gerede DM, Gulec S, Kilickap M, Kaya CT, Vurgun VK, Ozcan OU, Goksuluk H, Erol C. Comparison of a qualitative measurement of heart-type fatty acid-binding protein with other cardiac markers as an early diagnostic marker in the diagnosis of non-ST-segment elevation myocardial infarction. CVJA. 2015;26:204–209. doi:10.5830/CVJA-2015-028.
7. Schuchert A, Hamm C, Scholz J, Klimmeck S, Goldmann B, Meinertz T. Prehospital testing for troponin T in patients with suspected acute myocardial infarction. Am Heart J. 1999;138:45–48. doi:10.1016/S0002-8703(99)70244-9.
8. Sorensen JT, Terkelsen CJ, Steengaard C, Lassen JF, Trautner S, Christensen EF, Nielsen TT, Botker HE, Andersen HR, Thygesen K. Prehospital troponin T testing in the diagnosis and triage of patients with suspected acute myocardial infarction. Am J Cardiol. 2011;107:1436–1440. doi:10.1016/j.amjcard.2011.01.014.
9. Venturini JM, Stake CE, Cichon ME. Prehospital point-of-care testing for troponin: are the results reliable? Prehosp Emerg Care. 2013;17:88–91. doi:10.3109/10903127.2012.717166.
10. Stengaard C, Sorensen JT, Ladefoged SA, Christensen EF, Lassen JF, Botker HE, Terkelsen CJ, Thygesen K. Quantitative point-of-care troponin T measurement for diagnosis and prognosis in patients with a suspected acute myocardial infarction. Am J Cardiol. 2013;112:1361–1366. doi:10.1016/j.amjcard.2013.06.026.
11. van Dongen DN, Tolsma RT, Fokkert MJ, Badings EA, van der Sluis A, Slingerland RJ, van 'T Hof AW, Ottervanger JP. Pre-hospital risk assessment in suspected non-ST-elevation acute coronary syndrome: a prospective observational study. Eur Heart J Acute Cardiovasc Care. 2018; 1–8. doi:10.1177/2048872618813846.
12. Ezekowitz JA, Welsh RC, Weiss D, Chan M, Keeble W, Khadour F, et al. Providing rapid out of hospital acute cardiovascular treatment 4 (PROACT-4). J Am Heart Assoc. 2015;4:1–9.
13. Thygesen K, Alpert JS, Jaffe AS, Simoons ML, Chaitman BR, White HD, Joint ESCAAHAWHFTFftUDoMI. Third universal definition of myocardial infarction. Circulation. 2012;126:2020–2035.
14. Simel DL, Samsa GP, Matchar DB. Likelihood ratios with confidence: sample size estimation for diagnostic test studies. J Clin Epidemiol. 1991;44:763–770. doi:10.1016/0895-4356(91)90128-V.
15. Bruins Slot MH, van der Heijden GJ, Stelpstra SD, Hoes AW, Rutten FH. Point-of-care tests in suspected acute myocardial infarction: a systematic review. Int J Cardiol. 2013;168:5355–5362. doi:10.1016/j.ijcard.2013.08.002.
16. van der Laarse A, Cobbaert CM, Gorgels AP, Swenne CA. Will future troponin measurement overrule the ECG as the primary diagnostic tool in patients with acute coronary syndrome? J Electrocardiol. 2013;46:312–317. doi:10.1016/j.jelectrocard.2013.02.007.
17. Christenson RH, Mullins K, Duh SH. Validation of high-sensitivity performance for a United States Food and Drug Administration cleared cardiac troponin I assay. Clin Biochem. 2018;56:4–10. doi:10.1016/j.clinbiochem.2018.05.004.

PROOF

18. Pecoraro V, Banfi G, Germagnoli L, Trenti T. A systematic evaluation of immunoassay point-of-care testing to define impact on patients' outcomes. Ann Clin Biochem. 2017;54: 420–431. doi:10.1177/0004563217694377.
19. Palamalai V, Murakami MM, Apple FS. Diagnostic performance of four point of care cardiac troponin I assays to rule in and rule out acute myocardial infarction. Clin Biochem. 2013;46:1631–1635. doi:10.1016/j.clinbiochem.2013.06.026.
20. Stopyra JP, Harper WS, Higgins TJ, Prokesova JV, Winslow JE, Nelson RD, et al. Prehospital modified HEART score predictive of 30-day adverse cardiac events. Prehosp Disaster Med. 2018;33:58–62. doi:10.1017/S1049023X17007154.
21. van Dongen DN, Fokkert MJ, Tolsma RT, Badings EA, van der Sluis A, Slingerland RJ, van 'T Hof AWJ, Ottervanger JP. Value of prehospital troponin assessment in suspected non-ST-elevation acute coronary syndrome. Am J Cardiol. 2018; 122:1610–1616. doi:10.1016/j.amjcard.2018.07.037.
22. Guangquan L, Hualan H, Xin N, Yong H, Haolan S, Tongxing L, Baoxiu G, Hu N, Guixing L. Time from symptom onset influences high-sensitivity troponin T diagnostic accuracy for the diagnosis of acute myocardial infarction. Clin Chem Lab Med. 2016;54:133–142. doi:10.1515/cclm-2014-0776.
23. Aldous S, Pemberton C, Richards AM, Troughton R, Than M. High-sensitivity troponin T for early rule-out of myocardial infarction in recent onset chest pain. Emerg Med J. 2012; 29:805–810. doi:10.1136/emermed-2011-200222.

PROOF ONLY
