# Supplementary material for: Prehospital use of a modified HEART Pathway and point-of-care troponin to predict cardiovascular events
Source: PLoS One. 2020 Oct 7;15(10):e0239460. doi: 10.1371/journal.pone.0239460 (PMC7540888; doi:10.1371/journal.pone.0239460)
Supplement: S3 File — (DOC) [file pone.0239460.s003.doc]

**
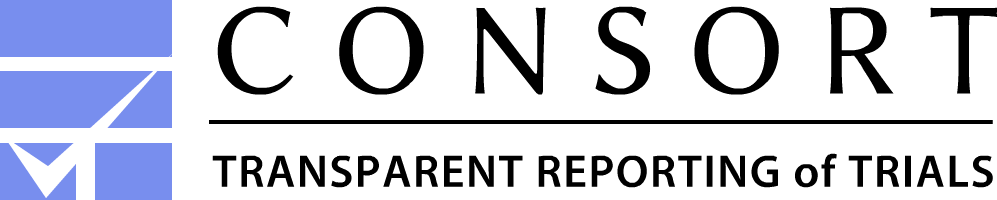
**

**CONSORT 2010 Flow Diagram**

**Allocation**

**Analysis**

**Follow-Up**

**Enrollment**

Assessed for eligibility (n= 506)

Excluded (n= 111)

♦  Not meeting inclusion criteria (n= 0)

♦  Declined to participate (n= 0)

♦  No PMHP assessment (n= 111)

Lost to follow-up (unable to contact or proof of life) (n= 18)
Discontinued intervention (give reasons) (n= 0)

Allocated to intervention (n= 395)

♦ Received allocated intervention (n= 395)

♦ Did not receive allocated intervention (give reasons) (n= 0)

Analysed (n= 395)
♦ Excluded from analysis (n= 0)

Randomized (N/A)
