# Supplementary material for: Prehospital use of a modified HEART Pathway and point-of-care troponin to predict cardiovascular events
Source: PLoS One. 2020 Oct 7;15(10):e0239460. doi: 10.1371/journal.pone.0239460 (PMC7540888; doi:10.1371/journal.pone.0239460)
Supplement: S4 File — (DOCX) [file pone.0239460.s004.docx]

**Can Pre-Hospital Use of the HEART Score and Abbott i-STAT^®^ Point-of-Care Troponin Predict Major Adverse Cardiovascular Events: the PARA-HEART Pilot Implementation**

Short Title: **PARA-HEART Pilot Implementation**

A Clinical Implementation Pilot Project

Principle Investigator: Simon A. Mahler, MD, MS

Co-Investigators:

Jason P. Stopyra, MD

James Winslow, MD

Robert D. Nelson, MD

Gregory Russell, MS

Gregory Pomper, MD

Chadwick D. Miller, MD, MS

Roy Alson, MD, PhD

David Zhao, MD

Consultants:

Robert F. Riley, MD, MS

**Table of Contents**

Section Page(s)

**A. Introduction 4-8**

**A1. Abstract 4-7**

**A2. Primary Hypothesis 7**

**A3. Purpose of the Protocol 8**

**B. Background 8-9**

**B1. Prior Literature and Studies 8-9**

**C. Primary Aims 9**

**D. Design 10-14**

**D1. Design Summary 10**

**D2. Setting 10-11**

**D3. Subject Selection and Withdrawal 11-12**

**D4. Quality Surveillance Design 12-14**

**E. Procedures 14-17**

**E1. Clinical Data Collection 14-15**

**E2. Outcomes 15-16**

**E3. Detection 17**

**E4. Endpoint Adjudication 17**

**F. Statistical Plan 17-18**

**F1. Overview 17**

**F2. Sample Size and Power 17**

**F3. Analysis Plan and Statistical Methods 17-18**

**F4. Missing Outcome Data 18**

**G. Data Handling and Record Keeping 18-19**

**G1. Confidentiality and Security 18**

**G2. Data Handling 18-19**

**G3. Training and Quality Assurance 19**

**G4. Case Report and Source Documents 19**

**G5. Standardization of Measurements 19**

**G6. Performance Monitoring 19**

**H. Data Quality Monitoring 19**

**H1. Data Quality Monitoring Plan 19**

**I. Recruitment and Timeline 20**

**J. Protection of Human Subjects 20-27**

**J1. Risks to Human Subjects 20-24**

**J2. Adequacy of Protection against Risks 24-26**

**J3. Potential Benefits of the Proposed Research to Subjects 26**

**and Others**

**J4. Importance of the Knowledge to be Gained 26**

**J5. Data and Safety Monitoring Plan 27**

**K. Attachments 28-29**

**K1. HEART Score Sheet 28**

**K2. Laboratory Method Validation and Quality Control 29**

**L. References 30-31**

**A. Introduction**

A1. Abstract

**Background**:

Approximately 8-10 million patients complaining of chest pain present to an Emergency Department (ED) annually in the United States. These patients are a challenge to healthcare providers, who are tasked with determining whether these symptoms are due to an acute coronary syndrome (ACS) or a non-ACS cause. Missing the diagnosis of ACS is associated with high rates of morbidity, mortality, and malpractice claims. Therefore, to avoid missing the diagnosis of ACS, patients with chest pain typically undergo extensive evaluations at an estimated cost of $10-13 billion annually. However, less than 10% of these patients are ultimately diagnosed with ACS. As the US healthcare system shifts towards a value-based model, it is clear that the current care patterns for acute chest pain, which fail to focus health system resources, such as hospitalization and cardiac testing, on patients most likely to benefit, are not sustainable.

In an effort to improve the quality and value of care for patients with acute chest pain, our group recently validated a risk stratification pathway, the HEART Pathway, which is designed to focus cardiac testing and admissions on higher-risk patients, who are more likely to benefit from testing. The HEART Pathway, which utilizes an easy to use clinical decision aid (the HEART score) and serial troponin measurement, has been shown to significantly reduce objective cardiac testing (stress testing and coronary angiography), shorten hospital length of stay, and increase the early discharge rate from the Emergency Department among patients with acute chest pain. These important efficiency gains occur without missing ACS and without increasing return visits to the ED or downstream admissions to the hospital over a 30 day period.

While the HEART score has been well validated in the ED setting, it has yet to be implemented in a prehospital setting. Paramedics are often the first providers to evaluate and begin treating patients with symptoms concerning for ACS. Current pre-hospital risk stratification is largely based on a combination of ECG results and paramedic gestalt. Since ECGs without signs of a STEMI are poor predictors of ACS, paramedics often have difficulty distinguishing high-risk patients from low-risk patients. Therefore, integrating objective risk stratification tools, such as the HEART score and point-of-care troponin testing, into Emergency Medical Services (EMS) triage and destination plans represents an opportunity to improve care. Furthermore, expanding use of the HEART score to paramedics in the pre-hospital setting is a natural extension of our prior work, especially given the growing sophistication of mobile integrated healthcare (“community paramedicine”) over the last decade. Thus, **multidisciplinary leaders within Emergency Medicine, Cardiology, and Prehospital Medicine have agreed that a limited/pilot evaluation of the HEART Score with Abbott’s i-STAT^®^ point-of-care (POC) troponin testing in the prehospital setting is needed.**

We anticipate that a standardized approach to paramedic risk stratification using the HEART score with Abbott’s i-STAT^®^ POC troponin testing will be feasible and achieve high accuracy for the detection of ACS. Ultimately we believe this planned implementation will improve the quality and value of chest pain care. Placing these tools in the hands of our first responders will identify patients with ACS earlier and speed the delivery of potentially life-saving care. For example, EMS triage and destination plans (chest pain treatment and transportation triage and destination plans) could be amended so that patients with positive POC troponins or high HEART scores could be transported directly to a hospital with cardiac catheterization capabilities, avoiding delays and costs associated with inter-facility transfers. However, before EMS triage and destination plans can fully incorporate the HEART score and POC troponin testing, first the feasibility of such an implementation must be established by collecting quality surveillance data.

To establish the feasibility and accuracy of HEART score and POC testing in the prehospital setting we propose a quality surveillance study of a limited implementation of the HEART score with POC troponin testing. This pilot will include paramedics from three demographically distinct counties (Forsyth, Surry, and Stokes counties) in North Carolina, who will begin using the HEART score and i-STAT POC Troponin as part of their risk assessment for patients with acute chest pain. However, EMS triage and transportation plans will not be altered based on the HEART score assessment until feasibility and accuracy have been established. To ensure the feasibility and accuracy of paramedic chest pain risk assessment we will be performing surveillance of electronic health records (EHR) and contacting patients by phone (which is a common practice in EMS quality assurance). The long term goal of this project is to provide data to support a larger scale implementation with greater integration of the HEART score and POC troponin testing into EMS triage and destination plans. Once the feasibility and accuracy are established, we anticipate that the HEART Score and POC troponin measurement will be used to speed the delivery of high-value care to patients with acute chest pain. While this project is a clinical pilot, given our intent to publish the data generated from this implementation, it meets the definition of human subjects research.

**Objective:**

The primary concept of the proposed quality improvement and research program is to assess the feasibility and accuracy of pre-hospital chest pain risk stratification by paramedics using the HEART Score and i-STAT POC troponin assay. In addition, we will determine the agreement and correlation between paramedic and ED physician risk stratification.

**Specific Aims:**

- **Specific Aim 1:** Perform validation (quality control) of the Abbott i-STAT^®^ point-of-care troponin assay in an ambulance; comparing troponin measurements from the patient’s blood samples analyzed in a moving ambulance vs a laboratory setting.
- **Specific Aim 2**: Determine whether paramedics using the HEART score and Abbott’s i-STAT^®^ point-of-care troponin assay in the pre-hospital setting can achieve 98% sensitivity and negative predictive value for major adverse cardiac events (MACE: cardiac death, myocardial infarction, or coronary revascularization) during the index visit and at 30 days in patients with symptoms concerning for ACS.
- **Specific Aim 3:** Test whether the inter-rater reliability (kappa) for risk assessment using the HEART score and Abbott’s i-STAT^®^ point-of-care troponin assay between ED physicians and paramedics exceeds 0.60.
- **Specific Aim 4:** Determine the potential impact of prehospital HEART Score and Abbott i-STAT^®^ point-of-care troponin measurement on the following efficiency outcomes: ED length of stay (LOS), hospitalization rate, and rate of objective cardiac testing (stress testing and coronary angiography).
- **Specific Aim 5**: Determine whether pre-hospital Abbott i-STAT^®^ point-of-care troponins are independent predictors of MACE during index hospitalization and at 30 days in patients with symptoms concerning for ACS.

**Methods:**

To improve the quality of care for patients with acute chest pain within Forsyth, Surry, and Stokes Counties, county EMS medical directors (Drs. Alson, Stopyra, and Nelson) as well as the EMS Medical Director for the state of North Carolina (Dr. Winslow) support a pilot implementation of the HEART Score including POC troponin testing beginning in 2016. To ensure the feasibility and safety of this implementation we propose a quality surveillance study. In addition, we have procured funding (Abbott Laboratories) which will support training of paramedics, supply the POC devices, and support the necessary quality surveillance for this implementation.

Over 70 paramedics will be trained to use the Abbott i-STAT device for POC troponin analyses and to calculate a HEART Score. Educational sessions will train the paramedics to correctly identify subjects appropriate for inclusion in the pilot and to correctly calculate a HEART Score. The educational tools will include in-person teaching and may include self-learning modules. Similar teaching methods that have been used in the HEART Pathway Implementation (PI Mahler) will be used in these sessions. Training regarding use of the Abbott i-STAT device will be conducted by Abbott Laboratories Inc. trainers. This will include the proper storage, maintenance, and use of device cartridges, quality assurance checks, and the interpretation and reporting or results. Training and competency events will be repeated during the pilot to maintain proficiency.

Following training, we will begin implementation in 18 ambulances across Forsyth County, Surry County, and Stokes County. Quality surveillance participants (n=500) will be identified retrospectively and quality assurance data will be collected electronically using EHR (EMS records, and WFBH health records) and via telephone follow-up calls (which are customary in EMS quality assurance projects). Eligible patients for this pilot implementation will be adult patients, over 21 years old, with non-traumatic chest pain or symptoms concerning for ACS, being transported to WFBMC ED. Patients with acute ST elevation myocardial infarction on initial pre-hospital ECG and patients being transferred from another hospital will be excluded. In addition, patients will be excluded when paramedics estimate that the patient’s pre-hospital time (scene time + transportation time) will be less than 5 minutes.

Paramedics will use a worksheet to obtain the historical and clinical data needed to calculate a HEART score. This will include performing POC troponin testing. This is within the scope of practice of paramedics who routinely perform venipuncture and POC testing on acutely ill patients. Furthermore, the feasibility of performing POC troponin analyses in a moving ambulance has been previously established. Given the paramedic’s need to use the POC troponin value for HEART score calculation they cannot be blinded to the troponin results. However, during this pilot, they will not deviate from their normal chest pain triage and destination plans based on the results. The routine chest pain triage and destination plans for these EMS counties include obtaining an IV, ECG, and the administration of aspirin, nitroglycerin, and supplemental oxygen. Also, during this pilot, i-STAT troponin and HEART Score results will not be used to determine or alter the patient’s transportation destination.

Since paramedics are not blinded to POC troponin results we will not prevent them from sharing their i-STAT troponin results with the ED care team as part of their typical transfer of care reports. However, they will do so using a study specific form, which clearly states that this POC troponin value is for study purposes only and is not to be used to determine a patient’s clinical care. We will train ED nurses and providers that EMS POC troponin results are part of a pilot, should not be acted on clinically at this time, and require confirmation with a core-lab troponin. While the ED care team will receive report on the POC troponin results (as well as the patient’s clinical features and ECG, as part of a normal EMS verbal report), the ED care team will remain blinded to paramedics’ HEART Scores to allow evaluation of agreement between paramedic and ED provider risk assessments. HEART Score calculation by the ED provider is part of the standard ED care for patients with acute chest pain presenting to the WFBMC ED, which will facilitate assessment for agreement. Paramedic POC troponin results and HEART Scores will not become part of the patient’s medical record.

Once participants have been transported to the WFBMC ED, they will receive a standard ED chest pain evaluation, expected to include an ECG and blood draws for troponin measurement. As part of normal care, blood obtained in the ED at presentation and 3 hours following presentation will be used for troponin measurement using the central lab [AccuTnI+3 assay (Beckman Coulter, California) or TnI-Ultra assay (Siemens, Munich Germany)]. In addition, most patients will also receive troponin measurement at presentation and 3 hours with an Abbott i-STAT POC assay.

In EMS quality assurance projects, it is customary for EMS agencies to contact their patients by phone to assess their well-being. Therefore, each patient will be contacted 30-90 days following their pre-hospital encounter to determine if they have had MACE (cardiac death, acute myocardial infarction, and coronary revascularization), hospitalizations, and objective cardiac testing (stress testing and coronary angiography). Patients will also have a record review to determine project outcomes including; MACE, hospitalization, hospital length of stay, and objective cardiac testing.

## A2. Primary Hypotheses

- Paramedics using the HEART score and Abbott’s i-STAT^®^ point-of-care troponin assay in the pre-hospital setting will achieve 98% sensitivity and negative predictive value for MACE during the index visit and at 30 days in patients with symptoms concerning for ACS.
- Inter-rater reliability (kappa) for HEART scores calculated by ED physicians in a hospital setting and paramedics in the pre-hospital setting using Abbott’s i-STAT^®^ point-of-care troponin assay will exceed 0.60.

##

## A3. Purpose of the Project Protocol

The objective of this pilot project is to establish the feasibility and accuracy of prehospital HEART score assessment using an Abbott’s i-STAT^®^ point-of-care troponin assay. If feasibility and accuracy can be established, the HEART score and POC troponin testing will allow modification of triage and destination plans so that patients can be transported to the most appropriate hospital settings as part of a larger implementation evaluation and to speed the delivery of ACS care. For example, patients with positive troponins and high HEART scores could be triaged to facilities with on-site cardiac catheterization laboratories, avoiding costly and inefficient inter-facility transfers. Furthermore, early risk stratification with the HEART Score, is likely to result in downstream efficiency gains; decreasing the ED length of stay for patients with low-risk chest pain.

**B. Background**

B1. Prior Literature and Studies

Coronary heart disease affects 15.8 million people in the US[^1^](#_ENREF_1) and represents a major public health dilemma**.** However, the evaluation process for this disease is inefficient. In the US, patients with symptoms of chest pain are generally evaluated in an ED. As a result, EDs care for 8-10 million patients annually with a complaint of chest pain.[^2^](#_ENREF_2)^,^ [^3^](#_ENREF_3) Of all patients presenting with chest pain, almost two thirds will be admitted to the hospital, but only 10% of these patients are ultimately diagnosed with an acute coronary syndrome (ACS).[^4-7^](#_ENREF_4) This over-triage has been estimated to cost $10-13 billion annually.[^8-10^](#_ENREF_8) Excessive admissions also contribute to hospital and ED crowding, which has been linked to an overall decrease in quality of care.[^11^](#_ENREF_11)^,^ [^12^](#_ENREF_12) This inefficiency also taxes the US in other ways such as lost productivity for affected patients and unnecessary invasive procedures. Equally important, this inefficiency consumes resources that could be diverted for other health interventions.

The evaluation of chest pain is also notoriously difficult for physicians. Despite a high rate of admissions for patients with chest pain, up to 4% of patients with acute myocardial infarction are inappropriately discharged from the ED.[^4^](#_ENREF_4) These patients, inadvertently discharged home with ACS, have higher mortality compared to those hospitalized[^4^](#_ENREF_4), emphasizing the importance of making the correct diagnosis the first time. Thus, physicians have adopted a strategy of admitting most patients with chest pain to observation units or inpatient beds for lengthy evaluations to avoid missing ACS. These practices lead to lengthy hospital stays, exacerbating hospital crowding, and increasing cost.

In response to this issue, a decision aid, called the HEART score was designed to risk stratify patients with chest pain or related symptoms without stress testing or cardiac imaging. It has five

components: History, Electrocardiogram (ECG), Age, Risk factors, and initial Troponin (**Table 1**).[^13^](#_ENREF_13)^,^ [^14^](#_ENREF_14) The HEART Score has been validated in over 5,000 patients, demonstrating a negative predictive value for MACE of 98.3% at 6 weeks.[^13-15^](#_ENREF_13) The HEART Pathway, which includes the HEART Score and serial troponin measurement at 0 and 3 hours increases negative predictive value to >99%.[^16-18^](#_ENREF_16) In a randomized control trial, funded by the American Heart Association, comparing the HEART Pathway to usual care we found that use of the HEART Pathway decreased objective cardiac testing at 30 days by 12.1% (68.8% vs 56.7%, p=0.048), length of stay in the hospital by 12 hours (9.9 vs 21.9 hours, p=0.013), and increased early discharges by 21.3% (39.7% vs 18.4%, p<0.001).

**Table 1.** The HEART score

Low-risk= ≤3, High-risk= ≥4

|  | | Points |
| --- | --- | --- |
| **H**istory | Highly Suspicious | 2 |
|  | Moderately Suspicious | 1 |
|  | Slightly Suspicious | 0 |
| **E**CG | Significant ST-depression | 2 |
|  | Non-specific repolarization abnormality | 1 |
|  | Normal | 0 |
| **A**ge | > 65 | 2 |
|  | 45-64 | 1 |
|  | < 45 | 0 |
| **R**isk factors | 3 or more risk factors | 2 |
|  | 1-2 risk factors | 1 |
|  | No risk factors | 0 |
| **T**roponin | > 3x normal limit | 2 |
|  | 1-3x normal limit | 1 |
|  | < normal limit | 0 |
| Total | |  |

No patients identified for early discharge had major adverse cardiac events at 30 days.[^18^](#_ENREF_18) Based on the results of this trial and the previous studies at WFBMC and in Europe, we have implemented the HEART Pathway at WFBMC. As of November 2014 the HEART Pathway has become the standard chest pain risk stratification tool used in the ED. The PARA-HEART project is a natural extension of our prior work with the HEART Pathway. Pre-hospital utilization of the HEART score has the potential to improve the accuracy of triage, speed care delivery to patients at high-risk of adverse events, and increase the efficiency of their care once they arrive at a medical facility.

## C. Primary Aims

- **Specific Aim 1:** Perform validation (quality control) of the Abbott i-STAT^®^ point-of-care troponin assay in an ambulance; comparing troponin measurements from patient’s blood samples analyzed in a moving ambulance vs a laboratory setting.
- **Specific Aim 2**: Determine whether paramedics using the HEART score and Abbott’s i-STAT^®^ point-of-care troponin assay in the pre-hospital setting can achieve 98% sensitivity and negative predictive value for major adverse cardiac events (MACE: cardiac death, myocardial infarction, or coronary revascularization) during the index visit and at 30 days in patients with symptoms concerning for ACS.
- **Specific Aim 3:** Test whether the inter-rater reliability (kappa) for risk assessment using the HEART score and Abbott’s i-STAT^®^ point-of-care troponin assay between ED physicians and paramedics exceeds 0.60.
- **Specific Aim 4:** Determine the potential impact of prehospital HEART Score and Abbott i-STAT^®^ point-of-care troponin measurement on the following efficiency outcomes: ED length of stay (LOS), hospitalization rate, and rate of objective cardiac testing (stress testing and coronary angiography).
- **Specific Aim 5**: Determine whether pre-hospital Abbott i-STAT^®^ point-of-care troponins are independent predictors of MACE during index hospitalization and at 30 days in patients with symptoms concerning for ACS.

Rationale for the Selection of Outcome Measures

In order for a chest pain risk stratification tool to be considered effective and achieve widespread adoption in the prehospital setting it must accurately detect patients with MACE and there must be high agreement between the paramedics and ED providers. While the HEART Score and troponin testing are well validated for risk stratification in the ED setting, feasibility and accuracy in the prehospital setting have yet to be tested as part of a clinical implementation. Furthermore, the inter-rater reliability (kappa) between paramedics and ED providers has not been tested.

Rationale for 30 day follow-up period

MACE outcomes will be assessed from the index visit through 30 days. ED ACS risk stratification evaluations have traditionally focused on 30 day end-points as recommended in the standardized reporting guidelines for ACS.[^19^](#_ENREF_19)

**D. Project design**

D1. Design Summary

We propose a quality surveillance study of HEART Score implementation, including point-of-care i-STAT troponin testing, within the Forsyth, Stokes, and Surry County Emergency Medical Services (EMS) systems. Once, training is completed, we will pilot an implementation of the prehospital HEART Score. The pilot implementation cohort will accrue 500 patients being transported to WFBMC ED over the course of 1 year. For quality surveillance activities, participants will be identified retrospectively and data will be collected electronically using EHR (EMS records, and WFBH health records) and via telephone follow-up calls (which are customary in EMS quality assurance projects). As part of this pilot we will validate the i-STAT troponin for EMS use. We will obtain prehospital i-STAT troponin measurements on all 500 patients and compare results to in-hospital measurements on the same sample. These POC measurements will not be used for clinical care.

Prior to implementation, over 70 paramedics will be trained to use the Abbott i-STAT device for POC troponin analyses and to calculate a HEART Score. Following completion of the educational sessions, we will begin implementation in 18 ambulances across Forsyth County, Surry County, and Stokes County. During this pilot, paramedics will deliver care via their normal chest pain triage and destination plans, which includes obtaining an IV, ECG, and the administration of aspirin, nitroglycerin, and supplemental oxygen. Troponin and HEART Score results will only be conducted on patients previously determined to be transported to WFBMC ED. Paramedics cannot be blinded to POC troponin results, but these results will not be used to determine or alter the destination hospital for transportation or to deviate from existing triage and destination plans. Paramedics will communicate POC troponin results to the ED staff at WFBMC using a study specific form, which clearly states that this POC troponin value is for study purposes only and is not to be used to determine a patient’s clinical care. We will train ED nurses and providers that EMS POC troponin results are part of a pilot, should not be acted on clinically at this time, and require confirmation with a core-lab troponin. Furthermore, we will emphasize that while prehospital troponin testing is meant to speed recognition of ACS it is not intended to supplant in-hospital testing. WFBMC ED attendings will be blinded to the paramedic’s HEART score to assess inter-observer agreement.

D2. Study Setting

To improve the quality of care for patients with acute chest pain within Forsyth, Surry, and Stokes Counties, county EMS medical directors (Drs. Alson, Stopyra, and Nelson) as well as the EMS Medical Director for the state of North Carolina (Dr. Winslow) support a prehospital pilot implementation of the HEART Score including POC troponin testing beginning in 2016. Forsyth County EMS has approximately 80 medics, 16 ambulances in service, and completes about 35,000 transports annually. Stokes County EMS has 34 medics, 5 ambulances, and completes 6000 transports each year. Surry EMS has 73 medics, 7 ambulances, and completes approximately 17,000 transports annually.

During this pilot we are limiting participation to patients transported to WFBMC. This project will not change EMS triage and destination plans to alter a patient’s transportation destination and will not change care once the patient arrives to WFBMC ED. The rationale for limiting inclusion to WFBMC patients is: a) it will allow greater oversight of paramedic activities, as all of the investigators involved are employed at WFBMC and all ED faculty and residents have been trained to calculate and interpret HEART Scores and b) it will enhance our ability to assess the accuracy of paramedics’ risk assessments, because index and follow-up data will be collected retrospectively from the electronic medical record (EMR). WFBMC is a tertiary care center with 821 licensed beds, full specialty and subspecialty availability, and full cardiac catheterization lab capability. The ED has 47 beds, is a level 1 trauma center for adults and pediatrics, and has an annual volume of >100,000 visits. The ED is staffed by board certified or board eligible emergency physicians 24 hours per day, 7 days a week who directly provide care and oversee care provided by residents and midlevel providers. The ED Clinical Decision Unit (CDU) is also open continuously, has 18 beds, and is staffed by midlevel providers and supervised by attending emergency physicians. The CDU operates on algorithm-driven care for a limited number of specified complaints such as chest pain. The only route of admission to the CDU is through the ED and the ED attending has ultimate responsibility for all decisions made in the CDU.

D3. Subject Selection and Withdrawal

**Inclusion Criteria**

1. Age greater than or equal to 21
2. Ambulance transport to WFBMC ED
3. Non-traumatic chest discomfort or other symptoms consistent with possible ACS
4. Patient being transported to WFBMC ED for further care

### Exclusion Criteria

1. ST-segment elevation in contiguous leads on any electrocardiogram (>/= 1 mV)
2. Inter-facility transfers
3. Short Pre-hospital times: Scene time + Transportation time anticipated to be less than 5 minutes.
4. Unstable vital signs: symptomatic hypotension at the time of enrollment (systolic < 90 mm Hg), tachycardia (HR>120), bradycardia (HR<40), and hypoxemia (<90% pulse-oximetry on room air or normal home oxygen flow rate)
5. Patient receiving hospice care
6. Concomitant non-cardiac medical, surgical, or psychiatric emergency

**Participation**

The target population is adult patients with acute chest pain or other symptoms suggestive of ACS, but without obvious ACS on ECG, who are being transported to WFBMC ED. Therefore, patients >21 years old with acute chest pain, without evidence of an ST-segment elevation myocardial infarction (STEMI) on ECG, will be included. Based on STEMI rates at WFBMC we expect less than 5% of patients with acute chest pain to be excluded due to ECG criteria.

This pilot has been selected for implementation based on collaborations across multiple disciplines including Emergency Medicine, Cardiology, and Prehospital Medicine. As a quality improvement project with broad-based support from local and state EMS systems, participation by human subjects will be part of customary care. Patients treated and transported for acute chest pain by paramedics in Forsyth, Surry, and Stokes County, specifically trained for this investigation, will have data abstracted from their EMS records. In EMS quality assurance projects, it is customary for EMS agencies to contact their patients by phone to assess their well-being. Therefore, each patient will be contacted 30-90 days following their pre-hospital encounter to determine if they have had MACE (cardiac death, acute myocardial infarction, and coronary revascularization), hospitalizations, and objective cardiac testing (stress testing and coronary angiography). Patients will also have index and outcome data abstracted from their WFBH EMR. These data will be reviewed to ensure the feasibility and accuracy of pre-hospital chest pain risk stratification by paramedics using the HEART score and i-STAT POC troponin assay. These quality assurance activities will be performed under a waiver of informed consent, obtained from the Institutional Review Board, as is currently utilized for similar quality surveillance programs at WFBH (i.e. the HEART Pathway Implementation).

### D4. Pilot Design

To ensure the feasibility and accuracy of prehospital chest pain risk stratification, using the HEART score and Abbott i-STAT POC troponin, we propose an **observational implementation design**. Since this pilot is observational it will not need a Clinical Laboratory Improvement Amendments (CLIA) certificate.

*i-STAT validation in moving ambulance:*, A validation of the i-STAT POC troponin assay in a moving ambulance will be performed. Previous study of the Abbott i-STAT POC troponin device showed high correlation between results in a moving ambulance compared with a laboratory environment. However, this is a small study and local validation is required before our paramedics and ED physicians use POC troponin results to influence clinical care. The first step of the i-STAT device validation will be to measure QC samples in a moving ambulance. Next, we will validate whole blood samples from all 500 patients by comparing i-STAT and central lab troponin results. These patients will have testing from the same blood draw with the Abbott i-STAT troponin assay in the ambulance followed by testing with the central lab assay: AccuTnI+3 assay (Beckman Coulter, California) or TnI-Ultra assay (Siemens, Munich Germany). The assay will be considered validated if the agreement between POC and central values (on “elevated” or “normal” results) in QC and patient samples is excellent; as defined by a kappa value >0.74. Validation will be done concurrent with the pilot implementation. The central lab troponin measurement will be run on whole blood obtained at the same time as blood used for the prehospital i-STAT. Please see Appendix 2 for the WFBMC Laboratory purposed method validation and quality control plan.*HEART Score Implementation Pilot:* Prior to implementation, over 70 paramedics will be trained to use the Abbott i-STAT device for POC troponin analyses and to calculate a HEART Score. Educational sessions will train the paramedics to correctly identify subjects appropriate for inclusion in the pilot and to correctly calculate a HEART Score. The educational tools will include in-person teaching and may include self-learning. Similar teaching methods and modules that have been used in the HEART Pathway Implementation (PI Mahler) will be used in these sessions. Training regarding use of the Abbott i-STAT device will be conducted by Abbott Laboratories Inc. trainers. This will include the proper storage, maintenance, and use of device cartridges, quality assurance checks, and the interpretation and reporting or results. In addition to initial training, Abbott will provide full training and competency support for 1 year. Training and competency events for all testing personnel will be conducted at start-up, at 6 months, and at 12 months. Documentation of training and competency will be completed. There is no intent to publish this quality control data.

Next, we will begin implementation in ambulances in Forsyth County, Surry County, and Stokes County. Eligible patients for this observational pilot implementation will be adult patients, over 21 years old, with non-traumatic chest pain or symptoms concerning for ACS, being transported to WFBMC ED. Patients with acute ST elevation myocardial infarction on initial pre-hospital ECG and patients being transferred from another hospital will be excluded. In addition, patients will be excluded when paramedics estimate that the patient’s pre-hospital time (scene time + transportation time) will be less than 5 minutes.

Paramedics will use a worksheet to obtain the historical and clinical data needed to calculate a HEART score. This will include performing POC troponin testing. This is within the scope of practice of paramedics who routinely start IVs and perform POC testing on acutely ill patients. Furthermore, the feasibility of performing POC troponin analyses in a moving ambulance has been previously established.[^20-22^](#_ENREF_20) While the paramedics will not be blinded to the troponin results, during this feasibility pilot, they will not deviate from their normal chest pain triage and destination plans based on the results of troponin testing or HEART score. The routine chest pain triage and destination plans for these EMS counties include obtaining an IV, ECG, and the administration of aspirin, nitroglycerin, and supplemental oxygen. Also, during this pilot, i-STAT troponin and HEART Score results will not be used to determine the destination hospital for transportation. The worksheet completed by the paramedics as part of this pilot will not be included in the patient’s permanent medical record. Paramedics will share i-STAT troponin results with the ED care team as part of their typical transfer of care reports, but will do so using a study specific form, which clearly states that this POC troponin value is for study purposes only and is not to be used to determine a patient’s clinical care. We will train ED nurses and providers that EMS POC troponin results are part of a pilot, should not be acted on clinically at this time, and require confirmation with a core-lab troponin.

*Rationale for not blinding paramedics and ED physicians to POC troponin results*: This is a clinical implementation pilot with a goal of testing the feasibility and accuracy of HEART score assessment using POC troponin results. A troponin result is a required component of the HEART score. Therefore in order to calculate a HEART score paramedics cannot be blinded to POC troponin results. Furthermore, through this implementation project we hope to gain greater understanding of the barriers and facilitators of a real-world implementation of the HEART score and POC troponin testing in a prehospital environment. Therefore, our implementation must closely match the expected paramedic workflow. In addition, we believe that asking unblinded paramedics to not share POC troponin results with receiving ED care team is unethical. In some patients POC troponin testing in the prehospital setting may provide early recognition of an acute myocardial infarction (non-ST-segment elevation myocardial infarction) and medics will be ethically compelled to share this information. However, since the POC troponin results are part of a pilot and their accuracy has not yet been confirmed, the paramedics will share results using a study specific form, which clearly states that this POC troponin value is for study purposes only and is not to be used to determine a patient’s clinical care. We will train ED nurses and providers that EMS POC troponin results are part of a pilot, should not be acted on clinically at this time, and require confirmation with a core-lab troponin. It is important to note that patients will already be receiving standard care for a possible NSTEMI. As part of their existing chest pain triage and destination plans paramedics will already be delivering standard care for acute myocardial infarction (aspirin, supplemental oxygen, and nitroglycerin). ED providers also routinely give patients with chest pain and possible NSTEMI aspirin, supplemental oxygen, nitroglycerin, and in high-risk cases use anticoagulation and antiplatelet therapy.

While the ED care team will receive report on the POC troponin results (as well as the patient’s clinical features and ECG, as part of a normal EMS verbal report), the ED care team will remain blinded to paramedics’ HEART Scores to allow evaluation of agreement between paramedic and ED provider risk assessments. HEART Score calculation by the ED provider is part of the standard ED care for patients with acute chest pain presenting to the WFBMC ED, which will facilitate assessment for agreement.

To assess the correlation of pre-hospital POC troponin to in hospital POC troponin and central lab troponin, patients in the WFBMC ED will receive a standard ED chest pain evaluation, expected to include an ECG and serial blood draw at ED arrival and 3 hours after arrival for troponin measurement. Blood obtained in the hospital as part of normal care will be used for central lab troponin measurement [AccuTnI+3 assay (Beckman Coulter, California) or TnI-Ultra assay (Siemens, Munich Germany)]. In addition, whenever possible, this blood will also be used to obtain an in-hospital Abbott i-STAT POC troponin measurement. See Table 2 for a summary of troponin measurements.

| Table 2. Troponin measures | | |  |
| --- | --- | --- | --- |
| **Blood Draw #** | **Description** | **i-STAT**  (green-top tube) | **Central lab/Beckmans**  (green-top tube) |
| 1 | Pre-hospital | X^*^ | X^#^ |
| 2 | ED arrival | X^#^ | X^#^ |
| 3 | 3 hours after ED arrival | X^#^ | X^#^ |

For quality assurance, participants will be identified retrospectively and data will be collected electronically using EHR (EMS records, and WFBH medical records) and via telephone follow-up calls. In EMS quality assurance projects, it is customary for EMS agencies to contact their patients by phone to assess their well-being. Therefore, each patient will be contacted 30-90 days following their pre-hospital encounter to determine if they have had MACE (cardiac death, acute myocardial infarction, and coronary revascularization), hospitalizations, and objective cardiac testing (stress testing and coronary angiography). Patients will also have a record review to determine patient demographics and project outcomes including; MACE, hospitalization, hospital length of stay, and objective cardiac testing.

## E. Project Procedures

## E1. Clinical Data Collection

For quality assurance data collection, participants will be identified retrospectively and data will be collected electronically using EHR (EMS records, and WFBH medical records) and via telephone follow-up calls.

Paramedics will use a worksheet to obtain the historical and clinical data needed to calculate a HEART score and this will include recording the POC troponin results. This worksheet will be an investigational record integrated into their normal reporting workflow. Data entered on this worksheet will be abstracted retrospectively. Data obtained will include demographic information (age, gender, race, ethnicity), date and time elements, characteristics of chest pain, ECG interpretation, cardiac risk factors (such as, prior coronary artery disease, smoking, hypertension, diabetes, hyperlipidemia, etc), POC troponin value, and the HEART score.

Since all participants will be transported to WFBMC ED, where the HEART Pathway is part of standard care, we will use the electronic medical record (EMR) to obtain ED physician HEART Score assessments and WFBMC troponin results. These data will be compared to paramedic assessments for determination of inter-observer agreement and correlation. We will also use the EMR to track index outcomes and 30 day events that occur within the WFBH system. Data collected from the WFBH EMR will include patient demographics, history and physical examination data, past medical history, cardiovascular risk factors, ECG results, laboratory results, HEART score and HEART Pathway assessments, ED and discharge diagnoses, disposition, index visit length of stay, objective cardiac testing at the index visit or within 30 days, recurrent ED visits, re-admissions, coronary revascularization, and MI or death within the follow-up period.

In addition to electronic surveillance, follow up phone calls will be used to determine outcomes at 30 days. EMS or project staff will contact each participant at 30-90 days following their pre-hospital encounter using a modified version of a previously described scripted follow-up dialogue[^23^](#_ENREF_23) to further clarify events since discharge, identify events occurring at other care facilities, and to determine health care utilization since discharge. Events of interest will include MACE (cardiac death, acute myocardial infarction, and coronary revascularization), hospitalizations, and objective cardiac testing (stress testing and coronary angiography), and recurrent ED visits. Participants still unable to be contacted will be considered censored at their last known contact unless record review reveals an event occurred, in which case the patient will be considered to have experienced an event. Participants unable to be contacted will be searched for in the Social Security Death Master File. In the event of discrepancy between a participant’s event reporting and the medical record, the medical record will be considered correct.

*Rationale for phone follow up:*  In order to determine the accuracy of paramedic HEART Score assessments for MACE we will need to have a low lost-to-follow-up rate. Without telephone follow-up calls patients who seek follow-up or recurrent care or have adverse events at non-WFBH facilities will not be detected. Furthermore, it is common practice for EMS agencies to use phone follow up to contact patients for quality assurance purposes. Using the above methods we expect to have a lost to follow-up rate less than 10%.

E2. Outcomes

*2a. Primary Outcome*

The primary outcome is MACE at 30 days. MACE is a composite outcome of cardiac death, acute myocardial infarction, and coronary revascularization. MACE occurring in patients with a low-risk HEART score assessment will be considered missed MACE. The primary outcome will be used to determine the sensitivity, negative predictive value, specificity, positive predictive value, likelihood ratios, and accuracy for paramedic detection of MACE at 30 days using the HEART score with i-STAT POC troponin testing.

The definition of cardiovascular death will be based on the Action to Control Cardiovascular Risk in Diabetes (ACCORD) trial.[^24^](#_ENREF_24) Death from stroke will not be considered a cardiac death.

Modified ACCORD definition of Cardiovascular Death:

1. Unexpected death: Unexpected death presumed to be due to ischemic cardiovascular

disease, occurring within 24 hours of the onset of symptoms without confirmation of

cardiovascular disease, and without clinical or post mortem evidence of other etiology.

2. Fatal Myocardial infarction (MI): death within 7 days of the onset of documented MI

3. Congestive heart failure (CHF): death due to clinical, radiological or postmortem

evidence of CHF without clinical or postmortem evidence of an acute ischemic event

(cardiogenic shock to be included).

4. Death after invasive cardiovascular interventions: death associated with the intervention,

i.e., within 30 days of cardiovascular surgery, or within 7 days of cardiac catheterization,

arrhythmia ablation, angioplasty, atherectomy, stent deployment, or other invasive

coronary or peripheral vascular intervention.

5. Documented arrhythmia: death due to bradyarrhythmias or tachyarrhythmias not

associated with an acute cardiac ischemic event.

6. Death following non-cardiovascular surgery: death due to cardiovascular causes as

defined above within 30 days of surgery.

The definition of myocardial infarction will be based on the recommendations of the 2012 Joint ESC/ACCF/AHA/WHF Task Force for the Redefinition of Myocardial Infarction:[^25^](#_ENREF_25)

A rise and/or fall of troponin with at least 1 value above the 99th percentile of the upper reference limit with at least one of the following: a) symptoms of ischemia, b) ECG changes indicative of new ischemia, c) Development of pathological Q waves on the ECG, d) Imaging evidence of new loss of viable myocardium or new regional wall motion abnormality. Peri-procedural MI is defined in accordance with the most recent 2012 Universal Definition of MI (Types 4a, 4b, and 5).

Coronary revascularization will be defined as angioplasty with or without stent placement, or coronary artery bypass surgery. Coronary revascularization occurring in patients discharged from the ED without objective cardiac testing will be considered missed coronary revascularization.

*2b. Secondary Outcomes*

Objective cardiac testing will be defined by any stress testing modality, coronary CT angiography, or invasive coronary angiography.

Length of stay (LOS) will be the time from ED arrival to hospital discharge for all patients, whether admitted or not.

Index visit hospitalization will be defined as an inpatient or observation admission (including Observation Unit evaluations ≥6 hours).

Cardiac related non-index hospital admissions and ED visits: Hospital admissions and ED visits occurring during the 30 day follow up period will be categorized as cardiac-related if a cardiac procedure is performed, the primary reason for admission/visit is chest pain, possible ACS, or a discharge diagnosis relates to chest pain, MI, acute coronary syndrome, heart failure, dysrhythmias, pericardial disease, or other cardiac disease. Cardiac procedures include cardiac imaging / stress testing (excluding resting echo), coronary revascularization, and pacemaker or defibrillator insertion.

### E3. Detection

MACE will be assessed at 30 days through the EMR and scripted phone follow up calls. Participants without EMR or phone follow up data will be searched for in the Social Security Death Master File.

## E4. Endpoint Adjudication

A consensus of two reviewers (Winslow, Stopyra) will adjudicate elements required to measure the occurrence of all MACE endpoints. Reviewers will adjudicate return visits to the ED or readmissions within the ED are cardiac related if this unclear based on preliminary review by project staff. To make these assessments, reviewers will be provided the participant’s index and discharge records, records obtained from follow-up including claims data when available, and project definitions. Records will have patient identifiers removed. Reviewers will complete a reviewer outcome form recording the occurrence of safety endpoints, which will be stored in our secured electronic database. Any disagreements will be settled by consensus between the two reviewers, or the involvement of a third reviewer (Nelson).

**F. Statistical Plan**

F1. Overview

The primary concept of the PARA-HEART project is to evaluate whether paramedics can be taught to calculate a HEART score accurately with acceptable intra-observer reliability (when compared to ED physician assessments). This project is a clinical pilot study in order to generate data to support a larger and non-observational implementation. If we are able to show that these scores can be reliably predict MACE and agree with ED physicians assessments, then we can start using the HEART score in the pre-hospital setting to alter care and transport triage and destination plans.

F2. Sample Size and Power

The PARA-HEART project is a clinical pilot study in which we hope to demonstrate that paramedics are able to achieve 98% sensitivity for the detection of MACE. To achieve a sensitivity of 98% with a lower bound of the 95% confidence interval that exceeds 90% we will need at least 50 patients with MACE events. Assuming a 10% MACE rate among our chest pain population,[^4-7^](#_ENREF_4) to include 50 patients with adverse events we will need a total sample size of 500 patients.

F3. Analysis Plan and Statistical Methods

The percentage of patients identified by paramedics using the HEART Score with POC troponin testing as high- and low-risk, the sensitivity, specificity, and positive and negative predictive values, positive and negative likelihood ratios, and accuracy for MACE will be calculated. Corresponding 95% exact binomial confidence intervals will be computed.

The proportion of patients receiving objective cardiac stress testing, cardiac catheterization, myocardial infarction, or cardiac death during the index hospitalization will be estimated for subjects classified as high and low risk by ED physicians and compared with those classified as high and low risk by paramedic assessment. Cardiac-related ED visits and readmission to the hospital will be calculated at 30 day follow-up. LOS will be reported using descriptive statistics, including median and inter-quartile range as this outcome tends to have a nonparametric distribution.

HEART scores calculated by the blinded primary ED provider will be compared to the HEART scores obtained by the paramedics. Raw percent agreement and kappa scores will be determined to assess intra-observer reliability. A kappa of >0.75 will be defined as excellent agreement, 0.60-0.74 will be defined as acceptable agreement, and <0.59 will be defined as unacceptable agreement.

*Post-Hoc Analysis of Troponin Cut Points:* For the clinical pilot, paramedics will use a cut point of 0.08 ng/mL, which corresponds to the published 99^th^ percentile upper reference limit for the assay. However, following completion of the pilot we will analyze different troponin cut points (including 0.04 ng/mL) used alone or in combination with the HEART score to find the combination with the greatest accuracy (area under the curve in a receiver operator curve).

## F4. Missing Outcome Data

Based on prior studies we expect >90% to have complete follow-up data from the EMR or phone follow up calls. Participants without follow up data will be searched for the Social Security Death Master File. Those with missing outcome data will be treated as censored and will be considered to have not had a safety event. A planed sensitivity analysis will be used to test this assumption.

**G. Data Handling and Record Keeping**

## G1. Confidentiality and Security

Electronic data will be housed on the secure server of the WFSM. The electronic database will contain the participant’s unique project identifier, medical record number, and full date elements, but will otherwise be clean of PHI. A key will be maintained linking the participant’s project ID number to their PHI, and will be stored by the WFSM, Department of Emergency Medicine in a locked office. Access to PHI will be restricted to the project team members, IRB, adjudication committee, and auditors. Data entry will be performed by trained research staff with valid confidentiality agreements.

## G2. Data Handling

Data will be processed using methods currently used by this project team. A detailed “sources of data” document describing the source of each data point will be created. Data collection templates will be integrated into REDCap. Validation rules will be applied to data as they are entered to reduce data queries. Automated reports will be generated displaying data quality assessments and form completion rates for discussion in Operations Meetings.

## G3. Training and Quality Assurance

A detailed manual of procedures (MOP) will be developed and contain definitions of all measurements and descriptions of all project procedures. This manual will be developed by the PI with assistance from the project staff. The MOP will be housed on a central server for project team access.

The research unit has implemented standard operating procedures for conducting clinical trials, specific to interacting with ED patients. The members of this research unit are trained on these procedures. The research unit has regularly occurring meetings to discuss vulnerable populations, recruitment progress, data quality, and workload.

The PI will train the primary coordinator on the project protocol and project procedures. The primary coordinator will then train other project team members. All project team members will be trained according to the Project Training List in the Manual of Procedures prior to project initiation.

## G4. Case Report Forms and Source Documents

Electronic case report forms will be used. Data whose original source is the electronic medical record will not be printed as it will be housed on the electronic medical record system of the hospital.

## G5. Standardization of Measurements

The manual of procedures will contain detailed descriptions of all project measurements. Standard definitions for measurement classifications have been chosen when available, and an adjudication committee will be used to determine more subjective measurements.

## G6. Performance Monitoring

Oversight of the project will be conducted by the PI. Data will be reviewed for completeness periodically by the PI. If data inconsistencies are noted, corrective actions will be taken.

**H. Data Quality Monitoring**

## H1. Data Quality Monitoring Plan

The PI will periodically review project data for quality and completeness. If data inconsistencies are noted, corrective actions will be taken and more frequent monitoring will occur.

Data tables will be examined by the PI / project staff periodically to determine missing data and to examine for implausible values. Electronic data queries will be generated for the project coordinator.

**I. Recruitment and Timeline**

Prior to beginning this pilot 3 months will be needed for training and project start-up. Following this start-up period, we anticipate a 12 month pilot project period will be required to obtain the 500 participants needed for this pilot. The pilot period will be followed by a 3 month close-out period to complete follow-up phone calls and record reviews.

| Project Timeline | | |
| --- | --- | --- |
| Summer 2016 | 2016-17 | 2017 |
| 3 months | 12 months | 3 months |
| Start-Up | Pilot Project | Close-Out |

**J. Protection of Human Subjects**

J1. Risks to Human Subjects

Overview: The HEART score with i-STAT POC troponin testing has been selected for local EMS system implementation based on collaborations across multiple disciplines including Emergency Medicine, Cardiology, and Prehospital Care. As a clinical quality improvement implementation with broad-based support, participation by human subjects will be part of customary care within the ambulance and upon arrival at WFBMC ED. Following implementation patients treated for acute chest pain by Forsyth, Stokes, and Surry County EMS and at WFBMC ED will have index and outcome data abstracted from an investigational pre-hospital record and the WFBMC EMR. In addition, as is customary in EMS quality surveillance patients will be contacted by phone to assess for adverse events. These data will be reviewed to ensure the feasibility and accuracy of a prehospital chest pain risk assessment using the HEART score with POC i-STAT troponin measurement. Quality surveillance activities will be performed under a waiver of informed consent, obtained from the Institutional Review Board, as is currently utilized for similar quality surveillance programs at WFBH (i.e. the HEART Pathway Implementation). Validation of thei-STAT troponin device in a moving ambulance will be completed concurrently by comparing POC and core-laboratory troponin results.

a. Human Subjects Involvement and Characteristics

The HEART Score pilot implementation has support from county and state EMS medical directors with a planned roll out in 2016. Planning is currently underway including coordination with the WFBH Point-Of-Care-Testing Committee and Abbott Laboratories Inc. Quality surveillance is being conducted to ensure the feasibility of this implementation (the accuracy and reproducibility of the HEART score as determined by paramedics in the field). Patients will receive standard care (which following implementation in 2016 will include HEART score calculation and POC troponin testing). Data will be collected from paramedic records, the WFBH EMR, and telephone follow-up calls. However, we are interested in publishing the findings of this implementation project. Therefore this project meets the definition of human subjects’ research.

For the pilot implementation we anticipate a sample size of approximately 500 subjects accrued over a 12 month period. Based on prior studies we expect about 90% to have complete follow-up data. We intend to exclude subjects who were less than 21 years old at the time of their ED visit, were having an acute ST-segment elevation MI (STEMI), or were inter-facility transfers. We anticipate this population will consist of an equal proportion of male and female patients of mean age 56 years based upon our preliminary data. Subjects will likely have increased cardiovascular risk factors compared to a normal population of controls. Subjects may have a history of cardiovascular disease. No special populations of subjects will be included. No collaborating sites will identify subjects. Specific inclusion and exclusion criteria are:

### Inclusion Criteria

1. Age greater than or equal to 21
2. Non-traumatic chest discomfort or other symptoms consistent with possible ACS
3. Patient being transported to WFBMC ED for further care

### Exclusion Criteria

1. ST-segment elevation in contiguous leads on any electrocardiogram (>/= 1 mV)
2. Inter-facility transfers
3. Short Pre-hospital times: Scene time + Transportation time anticipated to be less than 5 minutes.
4. Unstable vital signs: symptomatic hypotension at the time of enrollment (systolic < 90 mm Hg), tachycardia (HR>120), bradycardia (HR<40), and hypoxemia (<90% pulse-oximetry on room air or normal home oxygen flow rate)
5. Known terminal diagnosis with life expectancy less than 1 year
6. Concomitant non-cardiac medical, surgical, or psychiatric emergency

b. Sources of Materials

**Description of research material**

Research material obtained from the human subjects will include clinical data. Recorded data will include: demographic information, historical and physical exam data, laboratory results, radiologic and cardiac imaging results, diagnoses, clinical outcome information, all collected retrospectively.

**Description of data to be collected**

Data from the prehospital encounter, index hospital visit, and 30 day follow up period, including demographics, historical and physical exam data, laboratory results, radiologic and cardiac imaging results, diagnoses, length of stay, clinical outcome information and invasive procedures will be abstracted by the project team from EHR. Adverse events (MACE) will be identified by record review and by phone follow-up calls at 30-90 days after the index encounter.

**Access to individually identifiable private information**

Access to individually identifiable private information will be limited to those people required to access this information in order to conduct this project. This includes project team members, IRB, adjudication committee, and data monitors.

The project database will contain a limited amount of individually identifiable private information in the form of whole date elements and medical record numbers. Each participant will be assigned a unique sequential project identifier that will be linked to the participant’s research record and source documents through a log housed locally. Access to the entire project database will be limited to project team members at WFBMC who are involved in the trial management activities, including project team members, the institutional review board (IRB), adjudication committee, and data monitors.

**Collection, management, and protection of research specimens, records, and data Collection**

Data from the prehospital encounter, index visit, and 30 day follow up period, including patient demographics, history and physical examination data, past medical history, cardiovascular risk factors, ECG results, laboratory results, HEART score assessments, ED and discharge diagnoses, disposition, index visit length of stay, objective cardiac testing at the index visit or within 30 days, recurrent ED visits, re-admissions, coronary revascularization, and MI or death will be abstracted from the EHR and supplemented by phone follow-up calls. These data will be entered into a locally maintained and secured electronic database using the REDCap system. Validation rules will be applied to data as they are entered to reduce data queries. Automated reports will be generated displaying data quality assessments and form completion rates for discussion in Operations Meetings.

**Data Management**

The full electronic database will contain the participant’s unique project identifier, full date elements, and medical record numbers, but will otherwise be clean of identifiers. A key will link the project identifier to participants’ personal identifiers. This key will be housed in the project binder or electronically in a secure project folder behind the medical center firewall. Access to data containing personal identifiers will be restricted to the project team members, IRB, adjudication committee, and data monitors. Data entry will be performed by trained research staff with valid confidentiality agreements.

Our project team members have experience implementing an electronic process using a secure web-based data entry system for data collection and participant tracking. For this proposal, we will leverage forms and processes already in existence, along with our existing web infrastructure, REDCap.

The web-based system allows research personnel to interact with data using web forms. The website will be designed with input from the research staff so that the workflow follows the protocol and is in line with how the quality surveillance will be conducted. Website activity will be monitored and audited for security purposes. Users may view detailed tracking and management information for each participant and/or by assessment time point. Once logged in, research staff may run reports, enter data into forms, and review and edit data. As data are entered, validations rules are applied before data are saved. Inconsistencies are noted for staff to resolve. Research staff can resolve many queries immediately, comparing the screen to the source, often cleaning the entire database record on the spot. For queries not immediately resolvable, warnings are displayed whenever the data entry screen is recalled.

All access to the website will be logged and stored for auditing purposes.

**Data Protection - General**

Our security model for websites allows specification of access for each user to each area, allowing access to identifying information to be fully controlled. All research personnel are required to maintain current training in Protection of Human Subjects provided by Wake Forest School of Medicine or the CITI web program. All systems are securely controlled in the Biomedical Research Services Administration data center. Our core infrastructure consists of multiple servers, located in a secure network behind a firewall. Multiple intrusion detection systems monitor incoming and outgoing traffic patterns and signatures to identify and block potentially dangerous unauthorized attacks. Users are required to maintain secure passwords that expire every 90 days. Websites undergo a rigorous security scan by WFBMC’s Information Security department to ensure that users cannot be redirected to a third party site and to ensure that PHI information is secure.

**Data Protection - Disaster Recovery**

Each night, data, programs, code, documents, etc. associated with the project are backed up to a DLT tape library. These tapes are kept indefinitely and are located in a fireproof cabinet that remains locked at all times. Periodically, copies of tapes are moved to an off-site location for storage. In the event that there is any loss of data, the information can be restored from tape in a matter of hours.

c. Adverse Events, Unanticipated Problems, and Deviations

Any unanticipated problems, serious, and unexpected adverse events, deviations or protocol changes will be promptly reported by the principal investigator or designated member of the project team members to the IRB and sponsor or appropriate government agency if appropriate.

Expected adverse events in this project are limited to a potential loss of confidentiality. If a breach in confidentiality occurs, the event will be promptly reported to the IRB and other applicable regulatory agencies. The medical record will not be reviewed with the intent of detecting adverse events. In the event that an adverse event is identified during the chart review, the event will be recorded in the project records and IRB, sponsor, and other government agency event reporting criteria and guidelines will be followed.

d. Potential Risks

Project participants will receive standard/usual care and will be identified for participation in electronic quality surveillance retrospectively. For patients identified for participation in this project/quality surveillance, minimal risk is expected from participation beyond that encountered in daily clinical practice, as these participants will receive standard care. Patients with acute chest pain presenting to the ED are at risk of being discharged home with a major adverse cardiac event. Prior research on the HEART score and HEART Pathway here at WFBMC and in Europe on over 5,000 patients demonstrates that the HEART Pathway does not increase this risk. In fact, studies have documented ED ACS miss rates as high as 2-4% with usual care,[^4^](#_ENREF_4) so the HEART Pathway likely offers improvement in the detection of these high-risk patients.

**Risk of breach in confidentiality and privacy:** Given that all patients will receive standard care the primary risk of participation is a breach in privacy and confidentiality. We are collecting personal health identifiers as part of this research protocol, including medical record numbers, dates of birth, etc. Therefore, a potential risk to participants is breach in confidentiality and privacy. Safeguards are in place to prevent breach of confidentiality (see below) and therefore the likelihood of this occurring is small.

## J2. Adequacy of Protection against Risks

a. Recruitment and Consent

**Request of a waiver of consent:**

1. *The research involves no more than minimal risk to participants.* The risk of harm or discomfort that may occur as a result of taking part in this research is not expected to be more than in daily life or from routine physical or psychological examinations or tests. Patients identified for participation in this study/quality surveillance will receive standard care. Patients with chest pain already receive venipuncture as part of routine EMS care. EMS calculation of the HEART score and POC troponin testing are within the work scope of paramedics and should not add risk of harm or discomfort to participants. Furthermore, during this pilot project, which focuses on feasibility, the EMS treatment and transportation triage and destination plans will not be altered by HEART score or POC troponin results. Therefore, the primary risk of participation is a breach in privacy and confidentiality.
2. *The waiver of informed consent will not adversely affect the rights and welfare of the participants*. The rights and welfare of participants will be protected through the use of measures to maintain the confidentiality of project information.
3. *The research could not practicably be carried out without the waiver of informed consent.* To determine feasibility and accuracy of a prehospital HEART Score assessment, the HEART score and POC troponin testing must be utilized in a “real world” prehospital chest pain patient population. Performing informed consent would threaten the validity of this evaluation by introducing a significant selection bias. Furthermore, having paramedics obtain consent in the field is not practical or safe as this would prolong transport time and delay patient care, potentially leading to a worsening of clinical outcomes. In addition, ambulance transport for patients with acute chest pain is anxiety provoking. These patients are often concerned that they are having a heart attack and thus may not be in the appropriate frame of mind to ethically participate in informed consent during emergency transport.
4. *Whenever appropriate, the subjects will be provided with additional information after participation.* Project results will be presented or published (if possible) in lieu of providing individual subjects additional information regarding the project.

b. Protection against risk

**Risk of breach in confidentiality and privacy:**

This proposal will utilize our existing web infrastructure, REDCap. Electronic data being entered by research staff are immediately transmitted to a secure server, where data will reside throughout the project.

The full electronic database will contain the participant’s unique project identifier, medical record number, and full date elements, but will otherwise be clean of identifiers. A key will link the project identifier to participants’ personal identifiers. This key will be housed in the project binder or electronically in a secure project folder behind the medical center firewall. Access to data containing personal identifiers will be restricted to the project team members, IRB, adjudication committee, and data monitors. Data entry will be performed by trained research staff with valid confidentiality agreements.

The web-based system allows great flexibility in processing data management tasks, including monitoring and reporting. If needed, data can be securely downloaded directly by project team members for use in analyses.

The web-based system allows research personnel to interact with data using web forms. The website will be designed with input from the research staff so that the workflow follows the protocol and is in line with how the quality surveillance will be conducted. Website activity will be monitored and audited for security purposes. Users may view detailed tracking and management information for each participant and/or by assessment time point. Once logged in, research staff may run reports, enter data into forms, and review and edit data. As data are entered, validations rules are applied before data are saved. Inconsistencies are noted for staff to resolve. Research staff can resolve many queries immediately, comparing the screen to the source, often cleaning the entire database record on the spot. For queries not immediately resolvable, warnings are displayed whenever the data entry screen is recalled.

All access to the website will be logged and stored for auditing purposes. This system allows precise assignments for access based on the person’s role in the project. Once a user has successfully logged into the system, inactivity for a period of 30 minutes will automatically force the user to re-authenticate prior to using the system again.

Our security model for websites allows specification of access for each user to each area, allowing access to identifying information to be fully controlled. All research personnel are required to maintain current training in Protection of Human Subjects provided by Wake Forest School of Medicine or the CITI web program. All systems are securely controlled in the Biomedical Research Services Administration data center. Our core infrastructure consists of multiple servers, located in a secure network behind a firewall. Multiple intrusion detection systems monitor incoming and outgoing traffic patterns and signatures to identify and block potentially dangerous unauthorized attacks. Users are required to maintain secure passwords that expire every 90 days. Websites undergo a rigorous security scan by WFBMC’s Information Security department to ensure that users cannot be redirected to a third party site and to ensure that PHI information is secure.

Each night, data, programs, code, documents, etc. associated with the project are backed up to a DLT tape library. These tapes are kept indefinitely and are located in a fireproof cabinet that remains locked at all times. Periodically, copies of tapes are moved to an off-site location for storage. In the event that there is any loss of data, the information can be restored from tape in a matter of hours.

Safety Surveillance

The PI and project team members will monitor the quality surveillance results to ensure safety. All safety events will be reviewed by the PI and project team members.

## J3. Potential Benefits of the Proposed Research to the Subjects and Others

## Participants are not expected to experience any direct benefit. However, this pilot implementation may benefit society as a whole. This research will help determine the feasibility and accuracy of the HEART score assessment in the prehospital setting. Once feasibility and accuracy are established we believe greater integration of the HEART score and POC troponin testing into EMS triage and destination plans will lead to improved quality and value of care for patients with acute chest pain. In the future, this investigation could also lead to decrease hospital admissions, overcrowding, and improve health care efficiency.

## J4. Importance of the Knowledge to be Gained

This evaluation of paramedics’ ability to correctly calculate a HEART score using POC troponin testing will provide valuable data on its safety and efficacy as we move towards greater use in the pre-hospital setting. If the HEART score calculated by paramedics can be shown to have adequate sensitivity and correlation with physician scores, a large scale implementation in the pre-hospital setting could significantly improve outcomes and resource utilization in patients with acute chest pain.

## J5. Data and Safety Monitoring Plan

### 5a. Overview

The primary concept of the proposed pilot implementation is to evaluate whether paramedics accurately use the HEART score to calculate a patient’s risk for MACE with adequate intra-observer reliability. To accomplish this we will conduct quality surveillance on a planned pilot implementation of the HEART score and i-STAT POC troponin measurement in 3 local EMS systems. To ensure safety The PI and project team members will monitor the quality surveillance results. All safety events will be reviewed by the PI and project team members. Meetings by the PI and project team members and staff will occur routinely every 2-4 weeks. The IRB will be immediately notified if the risk: benefit ratio changes. Changes to the protocol and results of continuing annual review updates will be immediately reported to the IRB

### 5b. Monitoring entity

The PI and project team members will monitor the quality surveillance results.

### 5c. Procedures for monitoring project safety

When quality surveillance data becomes available it will be reviewed by the PI. The PI will communicate with the project team members and project staff to identify any safety concerns. Any possible adverse events will be discussed with the PI. Patient level data such as the results of individual testing will be reviewed by the PI during the conduct of the project. The PI will be responsible for ensuring that the protocol is conducted in compliance with all applicable elements of the Institutional Adverse Events and Unanticipated Problems Reporting and Management Policy. Changes to the protocol and results of continuing annual review updates will be immediately reported to the IRB.

### 5d. Procedures for minimizing research-associated risk

Steps taken to minimize research associated risk are itemized below by the risk.

Risk of breach in confidentiality and privacy: This proposal, will utilize our existing web infrastructure, REDCap. Please see section G for a complete discussion of the security and protection of data. Confidentiality agreements with participating insurers will be obtained.

### 5e. Procedures for protecting the confidentiality of participant data

Electronic data will be housed on the secure server of the WFSM. The electronic database will contain the participant’s unique project identifier, medical record number, and full date elements, but will otherwise be clean of PHI. A key will be maintained linking the participant’s project ID number to their PHI, and will be stored by the WFSM, Department of Emergency Medicine in a locked office. Access to PHI will be restricted to the project team members, IRB, adjudication committee, and auditors. Data entry will be performed by trained research staff with valid confidentiality agreements.

**K. Attachments**

K1. HEART Score Sheet


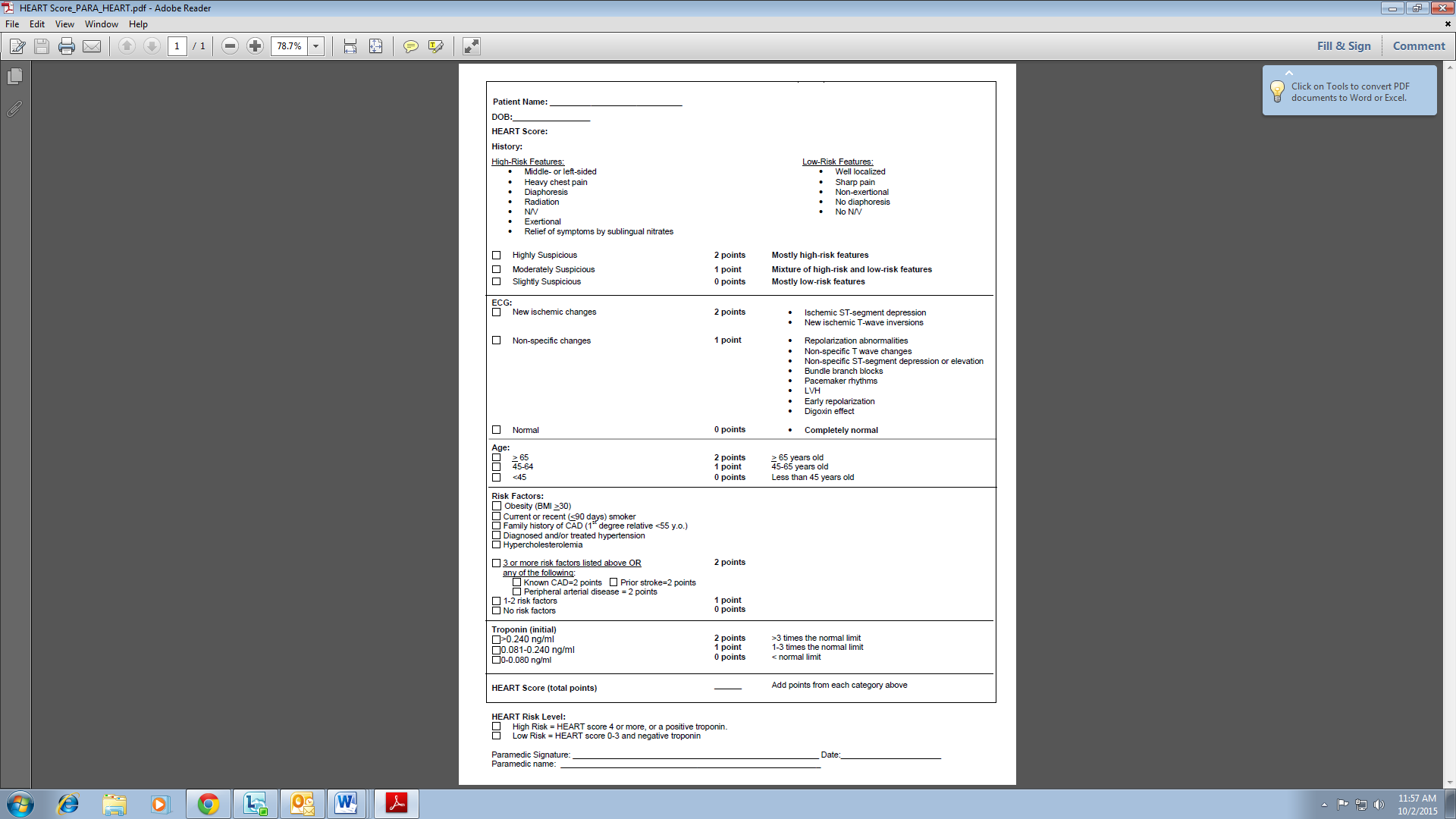


K2. Laboratory Method Validation and Quality Control

Wake Forest Baptist Medical Center

Point of Care Testing

Method Validation i-STAT Troponin CTNI Cartridge

**LINEARITY/AMR/REPORTABLE RANGE**

Analytical measurement range (AMR) verification includes 3 levels of calibration verification materials tested in triplicate.

- Reportable Range will be 0.00 to 35.00 ng/mL

**PRECISION**

Level 1, level 2, and level 3 liquid QC tested 20 times on the same day.

**Validation (Accuracy)**

- - - - 20 QC samples (level 1, 2, and 3) tested in a moving ambulance
- **Will occur concurrently with pilot implementation**

We will compare test samples between i-STAT and the Core Lab troponin method.

i-STAT tested from a FULL lithium heparin green top tube, collected in the field and tested immediately after collection. Blood will NOT be transferred from one collection container to another collection container. The SAME green top tube to be sent to the Core Lab for testing upon arrival to WFBH Emergency Department.

**REFERENCE RANGE STUDY:**

Will compare i-STAT results on patients with normal Core Lab troponins to verify published reference range.

**ANALYTICAL and FUNCTIONAL SENSITIVITY**

Information from Abbott

***Analytical and Functional Sensitivities***

- *The analytical sensitivity of the cTnI method is 0.02 ng/mL, which is the lowest cTnI level that can be distinguished from zero. The analytical sensitivity is defined as the concentration at two standard deviations from a sample at 0.00 ng/mL.*
- *Another characteristic of an analytical measurement is the functional sensitivity, which is defined as the cTnI level at which the test method displays a particular percent coefficient of variation (%CV). Estimates of the 20% and 10% functional sensitivity for the cTnI method were determined from whole blood measurements. The 20% and 10% functional sensitivities for the cTnI method are 0.07 ng/mL and 0.10 ng/mL, respectively*

**INDIVIDUALIZED QUALITY CONTROL PLAN**

An **Individualized Quality Control Plan** will be completed for the i-STAT troponin method.

- Once device is live, the test site should perform Level 1, level 2, and level 3 liquid QC in parallel with the daily internal electronic simulator for 20 days.
- After 20 days with successful QC checks, liquid QC will be decreased to 3 levels monthly and per each new shipment and per each new lot number.

**INTERFERING SUBSTANCES**

Manufacturer information evaluated for **Interfering Substances**

***Test Limitations***

*The frequency of suppressed results is affected by atmospheric pressure. Suppressed result rates may increase with higher elevations (decreased barometric pressure) and may become persistent if testing is performed at more than 7500 feet above sea level. Where unavailability of results is unacceptable, i-STAT recommends having an alternate test method available.*

*Samples from patients who have been exposed to animals or who have received therapeutic or diagnostic procedures employing immunoglobulins or reagents derived from immunoglobulins may contain antibodies, e.g., HAMA or other heterophile antibodies, which may interfere with immunoassays and produce erroneous results.18-24 The generation of potentially interfering antibodies in response to bacterial infections has been reported.16 While this product contains reagents that minimize the effect of these interferents and QC algorithms designed to detect their effects, the possibility of interference causing erroneous results should be evaluated carefully in cases where there are inconsistencies in the clinical information. Results from the i-STAT cTnI assay should be considered in the context of the entirety of the available clinical information. Medical decisions should not be based on a single i-STAT measurement.14*

*Cardiac troponin may not appear in circulation for 4-6 hours following the onset of symptoms of MI. Consequently, a single negative result is insufficient to rule out MI. The use of a serial sampling protocol is recommended practice.11*

*The results of different troponin assays are not generally comparable: cTnI and cTnT are distinct molecules and results are not interchangeable, nor comparable. In addition, significant variation in absolute troponin values may be observed for a given patient specimen with different analytic methods.13*

*Partially clotted samples can result in elevated cTnI results above the reference range, as well as quality check code errors. To prevent this from occurring, upon drawing the whole blood sample into a heparinized collection tube, the sample should be inverted gently at least 10 times to ensure even dissolution of the heparin anticoagulant.*

*Grossly hemolyzed samples can cause a decreased alkaline phosphatase activity, resulting in decreased detection of cTnI, increased assay backgrounds, and/or quality check codes.*

*Hematocrits in the range of 0-65% PCV have been demonstrated not to affect results. Samples with hematocrit levels above this range have demonstrated increases in the test imprecision and quality check codes.*

*The analyzer must remain on a level surface with the display facing up during testing. Motion of the analyzer during testing can increase the frequency of suppressed results or quality check codes. A level surface includes running the handheld in the downloader/recharger.*

**REFER TO Cartridge and Test Information Sheet for Troponin for additional information.**

iSTATtroponinMethodValidationSUMMARY081715

**L. References**

1. Rosamond W, Flegal K, Furie K, Go A, Greenlund K, Haase N, Hailpern SM, Ho M, Howard V, Kissela B, Kittner S, Lloyd-Jones D, McDermott M, Meigs J, Moy C, Nichol G, O'Donnell C, Roger V, Sorlie P, Steinberger J, Thom T, Wilson M, Hong Y. Heart disease and stroke statistics--2008 update: A report from the american heart association statistics committee and stroke statistics subcommittee. *Circulation*. 2008;117:e25-146

2. McCaig L, Nawar E. National hospital ambulatory medical care survey: 2004 emergency department summary. Advance data from vital and health statistics; no 372. Hyattsville, md: National center for health statistics. 2006

3. Owens PL, Barrett ML, Gibson TB, Andrews RM, Weinick RM, Mutter RL. Emergency department care in the united states: A profile of national data sources. *Ann Emerg Med*. 2010;56:150-165

4. Pope JH, Aufderheide TP, Ruthazer R, Woolard RH, Feldman JA, Beshansky JR, Griffith JL, Selker HP. Missed diagnoses of acute cardiac ischemia in the emergency department. *N Engl J Med*. 2000;342:1163-1170

5. Pines JM, Isserman JA, Szyld D, Dean AJ, McCusker CM, Hollander JE. The effect of physician risk tolerance and the presence of an observation unit on decision making for ed patients with chest pain. *Am J Emerg Med*. 2010;28:771-779

6. Fleischmann KE, Goldman L, Johnson PA, Krasuski RA, Bohan JS, Hartley LH, Lee TH. Critical pathways for patients with acute chest pain at low risk. *J Thromb Thrombolysis*. 2002;13:89-96

7. Roger VL, Go AS, Lloyd-Jones DM, Adams RJ, Berry JD, Brown TM, Carnethon MR, Dai S, de Simone G, Ford ES, Fox CS, Fullerton HJ, Gillespie C, Greenlund KJ, Hailpern SM, Heit JA, Ho PM, Howard VJ, Kissela BM, Kittner SJ, Lackland DT, Lichtman JH, Lisabeth LD, Makuc DM, Marcus GM, Marelli A, Matchar DB, McDermott MM, Meigs JB, Moy CS, Mozaffarian D, Mussolino ME, Nichol G, Paynter NP, Rosamond WD, Sorlie PD, Stafford RS, Turan TN, Turner MB, Wong ND, Wylie-Rosett J. Heart disease and stroke statistics--2011 update: A report from the american heart association. *Circulation*. 2011;123:e18-e209

8. Goldstein JA, Gallagher MJ, O'Neill WW, Ross MA, O'Neil BJ, Raff GL. A randomized controlled trial of multi-slice coronary computed tomography for evaluation of acute chest pain. *Journal of the American College of Cardiology*. 2007;49:863-871

9. Farkouh ME, Smars PA, Reeder GS, Zinsmeister AR, Evans RW, Meloy TD, Kopecky SL, Allen M, Allison TG, Gibbons RJ, Gabriel SE, The Chest Pain Evaluation in the Emergency Room (CHEER) Investigators. A clinical trial of a chest-pain observation unit for patients with unstable angina. *N Engl J Med*. 1998;339:1882-1888

10. Heller GV, Stowers SA, Hendel RC, Herman SD, Daher E, Ahlberg AW, Baron JM, Mendes de Leon CF, Rizzo JA, Wackers FJ. Clinical value of acute rest technetium-99m tetrofosmin tomographic myocardial perfusion imaging in patients with acute chest pain and nondiagnostic electrocardiograms. *J Am Coll Cardiol*. 1998;31:1011-1017

11. Sprivulis PC, Da Silva JA, Jacobs IG, Frazer AR, Jelinek GA. The association between hospital overcrowding and mortality among patients admitted via western australian emergency departments. *The Medical journal of Australia*. 2006;184:208-212

12. Miro O, Antonio MT, Jimenez S, De Dios A, Sanchez M, Borras A, Milla J. Decreased health care quality associated with emergency department overcrowding. *Eur J Emerg Med*. 1999;6:105-107

13. Six AJ, Backus BE, Kelder JC. Chest pain in the emergency room: Value of the heart score. *Neth Heart J*. 2008;16:191-196

14. Backus BE, Six AJ, Kelder JC, Mast TP, van den Akker F, Mast EG, Monnink SH, van Tooren RM, Doevendans PA. Chest pain in the emergency room: A multicenter validation of the heart score. *Crit Pathw Cardiol*. 2010;9:164-169

15. Backus BE, Six AJ, Kelder JH, Mosterd A, Mast EG, Groenemeijer B, Tio R, Veldkamp R, Doevendans PA. A prospective validation of the heart score for chest pain patients at the er. *Eur Heart J*. 2011;32:952

16. Mahler SA, Miller CD, Hollander JE, Nagurney JT, Birkhahn R, Singer AJ, Shapiro NI, Glynn T, Nowak R, Safdar B, Peberdy M, Counselman FL, Chandra A, Kosowsky J, Neuenschwander J, Schrock JW, Plantholt S, Diercks DB, Peacock WF. Identifying patients for early discharge: Performance of decision rules among patients with acute chest pain. *Int J Cardiol*. 2013;168:795-802

17. Mahler SA, Hiestand BC, Goff DC, Jr., Hoekstra JW, Miller CD. Can the heart score safely reduce stress testing and cardiac imaging in patients at low risk for major adverse cardiac events? *Crit Pathw Cardiol*. 2011;10:128-133

18. Mahler SA, Riley RF, Hiestand BC, Russell GB, Hoekstra JW, Lefebvre CW, Nicks BA, Cline DM, Askew KL, Elliott SB, Herrington DM, Burke GL, Miller CD. The heart pathway randomized trial: Identifying emergency department patients with acute chest pain for early discharge. *Circulation. Cardiovascular quality and outcomes*. 2015

19. Hollander JE, Blomkalns AL, Brogan GX, Diercks DB, Field JM, Garvey JL, Gibler WB, Henry TD, Hoekstra JW, Holroyd BR, Hong Y, Kirk JD, O'Neil BJ, Jackson RE. Standardized reporting guidelines for studies evaluating risk stratification of ed patients with potential acute coronary syndromes. *Acad Emerg Med*. 2004;11:1331-1340

20. Venturini JM, Stake CE, Cichon ME. Prehospital point-of-care testing for troponin: Are the results reliable? *Prehospital emergency care : official journal of the National Association of EMS Physicians and the National Association of State EMS Directors*. 2013;17:88-91

21. Stengaard C, Sorensen JT, Ladefoged SA, Christensen EF, Lassen JF, Botker HE, Terkelsen CJ, Thygesen K. Quantitative point-of-care troponin t measurement for diagnosis and prognosis in patients with a suspected acute myocardial infarction. *Am J Cardiol*. 2013;112:1361-1366

22. Sorensen JT, Terkelsen CJ, Steengaard C, Lassen JF, Trautner S, Christensen EF, Nielsen TT, Botker HE, Andersen HR, Thygesen K. Prehospital troponin t testing in the diagnosis and triage of patients with suspected acute myocardial infarction. *Am J Cardiol*. 2011;107:1436-1440

23. Kline JA, Mitchell AM, Runyon MS, Jones AE, Webb WB. Electronic medical record review as a surrogate to telephone follow-up to establish outcome for diagnostic research studies in the emergency department. *Acad Emerg Med*. 2005;12:1127-1133

24. Havas S. The accord trial and control of blood glucose level in type 2 diabetes mellitus: Time to challenge conventional wisdom. *Archives of internal medicine*. 2009;169:150-154

25. Thygesen K, Alpert JS, Jaffe AS, Simoons ML, Chaitman BR, White HD, Katus HA, Lindahl B, Morrow DA, Clemmensen PM, Johanson P, Hod H, Underwood R, Bax JJ, Bonow RO, Pinto F, Gibbons RJ, Fox KA, Atar D, Newby LK, Galvani M, Hamm CW, Uretsky BF, Steg PG, Wijns W, Bassand JP, Menasche P, Ravkilde J, Ohman EM, Antman EM, Wallentin LC, Armstrong PW, Januzzi JL, Nieminen MS, Gheorghiade M, Filippatos G, Luepker RV, Fortmann SP, Rosamond WD, Levy D, Wood D, Smith SC, Hu D, Lopez-Sendon JL, Robertson RM, Weaver D, Tendera M, Bove AA, Parkhomenko AN, Vasilieva EJ, Mendis S. Third universal definition of myocardial infarction. *Circulation*. 2012;126:2020-2035
