# Supplementary material for: Prehospital use of a modified HEART Pathway and point-of-care troponin to predict cardiovascular events
Source: PLoS One. 2020 Oct 7;15(10):e0239460. doi: 10.1371/journal.pone.0239460 (PMC7540888; doi:10.1371/journal.pone.0239460)
Supplement: S1 Table — (DOCX) [file pone.0239460.s005.docx]

**S1 Table** Patient characteristics for the entire cohort, the 395 patients in the analysis set with completed prehospital modified HEART Pathway (PMHP) assessments, and the 111 patients without completed PMHP assessments.

| Patient Characteristic | Total  N=506 | Patients in Analysis Set N=395 | Patients NOT in Analysis Set N=111 | In vs. Out of Analysis Set |
| --- | --- | --- | --- | --- |
| Age years – mean ± SD | 58.6 ± 15.3 | 58.5 ± 15.2 | 59.1 ± 15.6 | P = 0.745 |
| Sex (female) | 258/506 (51.0%) | 210/395 (53.2%) | 48/111 (43.2%) | P = 0.069 |
| Race |  |  |  |  |
| Caucasian | 250/503 (49.7%) | 199/392 (50.8%) | 51/111 (46.0%) | P = 0.646 |
| African American | 226/503 (44.9%) | 173/392 (44.1%) | 53/111 (47.8%) |  |
| Asian | 4/503 (0.8%) | 3/392 (0.8%) | 1/111 (0.9%) |  |
| Native American | 2/503 (0.4%) | 1/392 (0.3%) | 1/111 (0.9%) |  |
| Other | 21/503 (4.2%) | 16/392 (4.1%) | 5/111 (4.5%) |  |
| Ethnicity (Hispanic) | 20/505 (4.0%) | 16/395 (4.1%) | 4/110 (3.6%) | P = 1.000 |
| Risk factors |  |  |  |  |
| Current smoking | 128/506 (25.3%) | 103/395 (26.1%) | 25/111 (22.5%) | P = 0.537 |
| Hypertension | 335/497 (67.4%) | 256/389 (65.8%) | 79/108 (73.2%) | P = 0.165 |
| Hyperlipidemia | 141/506 (27.9%) | 109/395 (27.6%) | 32/111 (28.8%) | P = 0.811 |
| Diabetes | 146/506 (28.9%) | 123/395 (31.1%) | 23/111 (20.7%) | **P = 0.033** |
| Family history of CAD | 118/506 (23.3%) | 97/395 (24.6%) | 21/111 (18.9%) | P = 0.253 |
| BMI >30 (kg/m^2^) | 232/492 (47.2%) | 181/383 (47.3%) | 51/109 (46.8%) | P = 1.000 |
| Prior coronary disease | 148/499 (29.7%) | 113/389 (29.1%) | 35/110 (31.8%) | P = 0.636 |
| Prior MI | 96/499 (19.2%) | 71/389 (18.3%) | 25/110 (22.7%) | P = 0.337 |
| Prior PCI | 76/498 (15.3%) | 61/388 (15.7%) | 15/110 (13.6%) | P = 0.655 |
| Prior CABG | 50/500 (10.0%) | 35/390 (9.0%) | 15/110 (13.6%) | P = 0.153 |
| Prior CHF | 68/499 (13.6%) | 54/389 (13.9%) | 14/110 (12.7%) | P = 0.875 |
| Prior PVD | 23/506 (4.6%) | 21/395 (5.3%) | 2/111 (1.8%) | P = 0.193 |
| Prior stroke | 47/506 (9.3%) | 41/395 (10.4%) | 6/111 (5.4%) | P = 0.138 |

| Patient Outcomes | Total  N=506 | Patients in Analysis Set N=395 | Patients NOT in Analysis Set N=111 | In vs. Out of Analysis Set |
| --- | --- | --- | --- | --- |
| 30-day MACE | 92/506 (18.2%) | 74/395 (18.7%) | 18/111 (16.2%) | P = 0.581 |
| 30-day Death/MI | 87/506 (17.2%) | 70/395 (17.7%) | 17/111 (15.3%) | P = 0.669 |
| Index MACE | 85/506 (16.8%) | 70/395 (17.7%) | 15/111 (13.5%) | P = 0.319 |
| Index Death/MI | 79/506 (15.6%) | 65/395 (16.5%) | 14/111 (12.6%) | P = 0.376 |
| Lost to Follow-up | 22/506 (4.4%) | 18/395 (4.6%) | 4/111 (3.6%) | P = 0.797 |
