# Supplementary material for: Prehospital use of a modified HEART Pathway and point-of-care troponin to predict cardiovascular events
Source: PLoS One. 2020 Oct 7;15(10):e0239460. doi: 10.1371/journal.pone.0239460 (PMC7540888; doi:10.1371/journal.pone.0239460)
Supplement: S2 Table — (DOCX) [file pone.0239460.s006.docx]

**S2 Table** Diagnostic characteristics for prehospital modified HEART Pathway and core-lab-prehospital modified HEART Pathway for index MACE and Death/MI

|  | | Index MACE | | Index Death/MI | |
| --- | --- | --- | --- | --- | --- |
|  |  | PMHP | Core-Lab-PMHP | PMHP | Core-Lab-PMHP |
| High Risk | Specificity (95%CI) | 96.6%  (94.0-98.3%) | 91.8%  (87.5-95.0%) | 96.7%  (94.1-98.3%) | 92.0%  (87.8-95.1%) |
|  | PPV  (95%CI) | 60.7%  (40.6-78.5%) | 64.8%  (50.6-77.3%) | 60.7%  (40.6-78.5%) | 64.8%  (50.6-77.3%) |
|  | +LR  (95%CI) | 7.175  (3.517-14.640) | 7.770  (4.833-12.494) | 7.846  (3.857-15.963) | 8.732  (5.469-13.941) |
| Low Risk | Sensitivity (95%CI) | 90.0%  (80.5-95.9%) | 100%  (93.5-100%) | 89.2%  (79.1-95.6%) | 100%  (92.9-100%) |
|  | NPV  (95%CI) | 94.4%  (88.7-97.7%) | 100%  (95.2-100%) | 94.4%  (88.7-97.7%) | 100%  (95.2-100%) |
|  | -LR  (95%CI) | 0.278  (0.136-0.569) | 0  (0-NA) | 0.304  (0.149-0.621) | 0  (0-NA) |
